# Seed Size Variation of Trees and Lianas in a Tropical Forest of Southeast Asia: Allometry, Phylogeny, and Seed Trait - Plant Functional Trait Relationships

**DOI:** 10.3389/fpls.2022.852167

**Published:** 2022-05-17

**Authors:** Pornwiwan Pothasin, Emmanuel Paradis, Warren Y. Brockelman, Anuttara Nathalang, Thantiyapawn Khemrugka, Noppawan Lomwong, Patcharaphan Thripob, Rampai Saenprasert, Wirong Chanthorn

**Affiliations:** ^1^Department of Environment Technology and Management, Faculty of Environment, Kasetsart University, Bangkok, Thailand; ^2^Conservation Biology Program, School of Interdisciplinary, Mahidol University, Kanchanaburi Campus, Kanchanaburi, Thailand; ^3^ISEM, IRD, Université Montpellier, CNRS, Montpellier, France; ^4^National Biobank of Thailand, National Science and Technology Development Agency, Pathum Thani, Thailand; ^5^Institute of Molecular Biosciences, Mahidol University Salaya, Nakhon Pathom, Thailand; ^6^Department of Ecological Modelling, Helmholtz Centre for Environmental Research – UFZ, Leipzig, Germany

**Keywords:** functional traits, leaf toughness, plant height, seed dispersal, successional niche, seasonal tropical forest, liana, leaf trait

## Abstract

Seed size is a key trait for understanding and predicting ecological processes in a plant community. In a tropical forest, trees and lianas are major components driving ecosystem function and biogeochemical processes. However, seed ecological research on both components remains limited, particularly phylogenetic patterns and relationships with other traits. Here, we compiled a unique dataset of seed size (seed mass and geometrical size metrics) based on collections of more than 5,200 seeds of 196 woody plant species, covering >98 and 70% of tree and liana stems, respectively, located on a 30-ha plot in a tropical evergreen forest in central Thailand. We aimed to (1) develop allometric equations among seed size metrics to predict seed mass; (2) examine phylogenetic influence on seed size variation; and (3) examine relationships among seed traits and several other functional plant traits. Our allometric equations relating seed mass, seed volume, and width were well-fitted with data (*R*^2^*=* 0.94, 0.87 respectively). A phylogenetic signal test found that seed size was randomly distributed across the phylogeny. To study the functional trait relationships, we separately tested seed size data of the tree and liana communities (146 and 50 species, respectively), against mean body size of frugivores, successional niches, leaf, and structural traits. For the tree community, seed size was significantly related to mean body size of frugivores, which we believe is a basic driver of seed size because it is related to the gape width affecting dispersal effectiveness. Nearly all leaf traits were significantly positively correlated with seed size (*p* < 0.03). The significant positive correlation of leaf area and greenness suggested the high-energy demand of large-seeded species. We found a strong positive correlation between seed size and leaf toughness, suggesting a coordination between seed size and leaf defense. However, all these patterns disappeared in the same analysis applied to the liana community. Liana seed size variation was lower than that of trees, perhaps because lianas grow in relatively more uniform conditions in the forest canopy. Frugivore size was the strongest driver of seed size variation. Our study shows a surprising contrast between trees and lianas that is worth further investigation.

## Introduction

Seed size (here is defined by mass or geometric size metrics, such as volume, length, width, and thickness) is a trait central to many aspects of plant ecology. It is a key trait for understanding and predicting ecological processes in the early phases of a plant’s life cycle, for example, dispersal, persistence or dormancy, germination, and establishment (Leishman et al., 2000; Fenner and Thompson, 2005; Moles et al., 2005; Saatkamp et al., 2019). Seed mass is a widely applied metric of seed size in many studies of plant functional traits (Moles, 2018). However, geometric metrics, such as seed volume and seed length, are measured in most studies relevant to dispersal ecology (De Jager et al., 2019) and animal foraging behavior (Yi and Wang, 2015). Often, both seed size metrics and seed mass are not reported in the same studies; hence, we do not have enough information to accurately interchange between them (Kitamura et al., 2002; Moles et al., 2005; Pérez-Harguindeguy et al., 2013; Bello et al., 2015; Díaz, et al., 2016). We propose to develop an allometric equation that can predict seed mass from seed size and vice versa. Generally, the best-fit allometric model is the relationship between seed mass and volume, whereas the relationship with geometric measures, such as seed length or shape, vary among studies (Brewer, 2001; Wright et al., 2007; Casco and Silva Dias, 2008; Wang and Chen, 2009; Ganhão and Dias, 2019). Furthermore, most studies are biased toward Neotropical forests (Wright et al., 2007).

Ecologists have sought to explain variation of seed size within and between plant communities (Coomes and Grubb, 2003; Moles et al., 2007), because variation in seed size is crucial for understanding traits, such as competitive ability (Leishman and Murray, 2001; Turnbull et al., 2004), tolerance to environmental stressors (Baker, 1972; Westoby, 1998), seed predation (Thompson, 1987), seed dispersal (Greene and Johnson, 1993), and dormancy (Harel et al., 2011). In a tree community, seeds of pioneer species are normally smaller than those of shade-tolerant species (Ghazoul and Sheil, 2010). Seed size variation between pioneer vs. shade-tolerant species needs to be reinvestigated based on an objective classification, and systematically assessed *via* the probability of species occurrence in second- versus old-growth forests (e.g., Chazdon et al., 2011).

In fleshy-fruited plants, seed dispersers are regarded as important drivers of seed size, especially in tropical forests, where >70–80% of plant species are dispersed by animals (Osuri et al., 2016; Chanthorn et al., 2019). Generally, the achievement of seed dispersal is determined by whether a seed can pass into the mouth of a frugivore and be taken away from a fruiting tree. A key disperser trait is gape width, which should be wider than seed width (Fleming et al., 1993). Although many physiological and ecological factors may produce selection on seed size, the availability of effective dispersers may place an upper limit on seed size. Strong evidence that gape width of frugivores exerts selective pressure on seed size comes from a study showing a reduction of seed size in Neotropical forests with high defaunation intensity of large-bodied dispersers compared to non-defaunated forests, a phenomenon that has occurred only within the last 100 years (Galetti et al., 2013). However, since gape width data are difficult to collect, body size is often used as a proxy. The study of “anachronisms,” for example, demonstrated the consequences of large animal defaunation during the Pleistocene era that affected the dispersal of ill-suited plants with very large seed size (Janzen and Martin, 1982; Pires et al., 2017; Lim et al., 2020). As this topic has been studied mostly in Neotropical forests (Fuzessy et al., 2018; Lim et al., 2020), studies from other tropical regions are needed to gain a global perspective on this subject.

Coordination (positive correlation) and trade-off (negative correlation) of functional traits are common features in plant communities (Díaz, et al., 2004; Moles, 2018), including tropical forests (Wright et al., 2010). Coordination between tree height and seed size has been reported in a global study (Díaz et al., 2016) and in a study focusing only on trees in Neotropical forests (Bello et al., 2015). For the trade-off, species with a high leaf area normally have lower wood density, which supports fast growth in high-light environments (Chave et al., 2009). Intuitively, large seeds tend to generate large seedlings and provide an adaptive advantage to seedlings growing in shade environments, but the relationship between seed and leaf size lacks direct causation and is predicted to be weaker compared to that between seed size and plant height (Díaz et al., 2016). Alternatively, another global study showed a strong correlation between seed size with leaf life span (Moles, 2018). Interestingly, a cross-continental study found a strong relationship between seed size and leaf toughness (fracture resistance), suggesting another functional trait coordination of importance (Kraft et al., 2015).

Here, we compiled data on seed size for a majority of species in a tropical forest community, which was challenging because of the irregular fruiting or masting of most tree species (Suwanvecho et al., 2017; Kurten et al., 2018). Lianas are equally important and typically comprise about one-third of woody plant diversity in tropical forests (Schnitzer, 2018). Studies of lianas have lagged those of trees, and only a few studies have examined seed size or mass in more than a minority of liana species (e.g., Gallagher and Leishman, 2012).

Here we collected a seed size dataset of >5,000 seeds measured in five metrics: seed mass, seed volume, seed length, seed width, and seed thickness, covering >98 and > 70% of tree and liana stems, including many liana species not previously reported in TRY global databases (see Supplementary Table 3). We have, in addition to the Mo Singto plot, which is covered with relatively old-growth forest, a chronosequence of smaller plots covering all successional stages in the same landscape, allowing us to classify all tree species into pioneer, shade-tolerant, and generalist types (Chazdon et al., 2011). The life-history traits available for analysis fall into four groups: leaf traits (leaf area, leaf toughness, specific leaf area, and leaf greenness), structural traits (maximum plant height and wood specific gravity), successional niche, and seed dispersal agents or mean body size of frugivores.

The objectives of our study were to create allometric equations among seed size metrics, to examine phylogenetic influence on seed size variation, and to evaluate the relationship (coordination or trade-off) between seed size and plant traits by testing four hypotheses: (1) Large seed size is coordinated with other plant traits that are associated with shade tolerance and longer leaf life span, as opposed to faster growth; (2) Large seed size is associated with old-growth forest as opposed to early successional forest; (3) seed size is correlated with disperser size, as effective dispersal is the initial major hurdle to overcome in a plant’s life history; and (4) trees and lianas are expected to show different seed size–life-history trait relationships, because although reproductive lianas occupy similar light environments in the forest crown, they have different growth trajectories and means of support. We compared models with and without phylogenetic correction to examine the independence of variables and perhaps gain insight related to evolution.

## Materials and Methods

### Study Site

**Figure 1:**
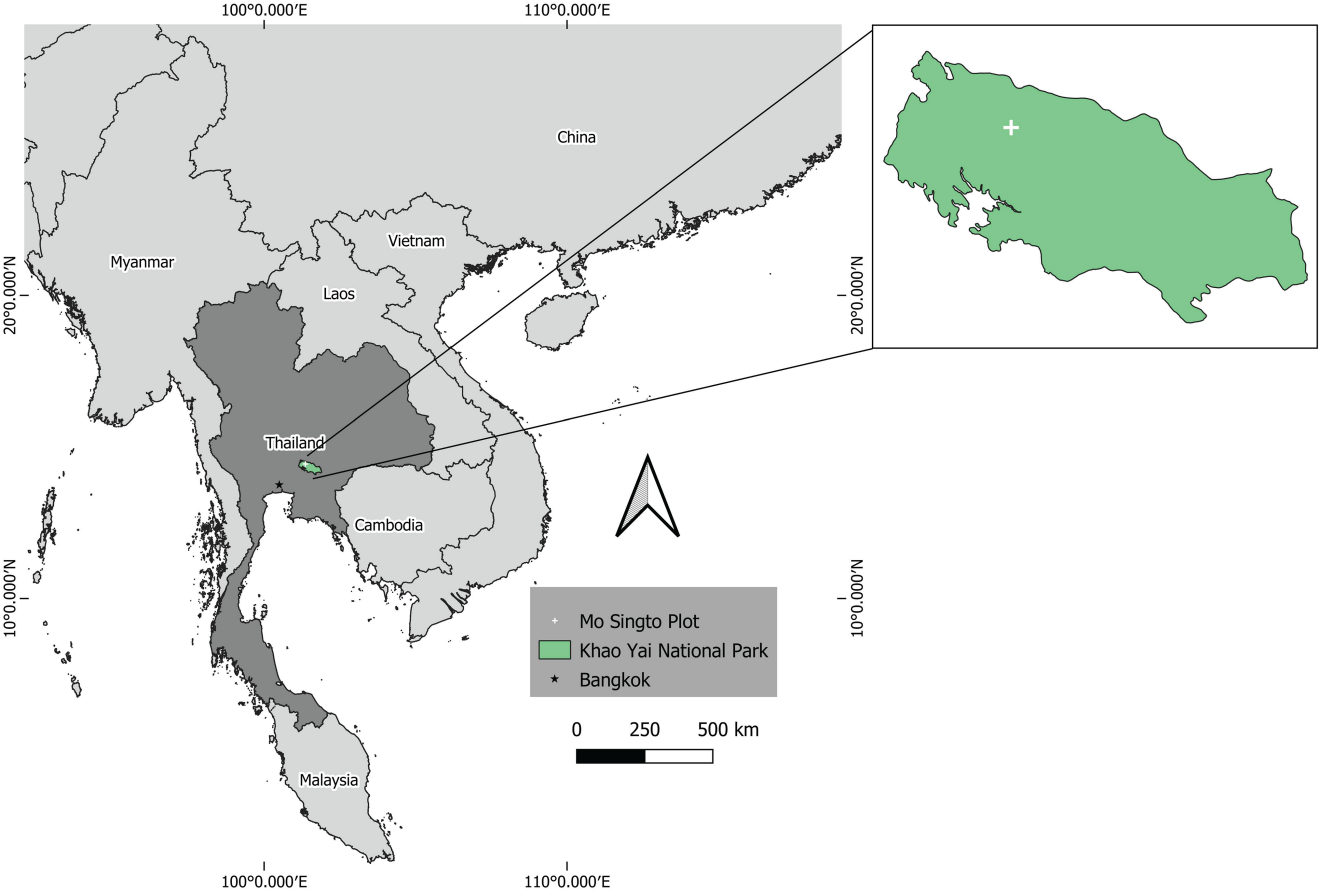
Map of Khao Yai National Park and the Mo Singto plot.

This study was carried out on the 30-ha Mo Singto forest dynamics plot (Brockelman et al., 2017; Figure 1), which is a member of the ForestGEO network of the Smithsonian Institution, Washington DC (Davies et al., 2021).^1^ The plot was established around 2001 in Khao Yai National Park (101°22′E and 14°26′N), a UNESCO world heritage site of Thailand. All trees with DBH ≥1 cm have been fully identified, tagged, and mapped. A re-census has been conducted every 5 years, the latest during 2020–2021. This plot is imbedded in a gibbon (*Hylobates lar*) study site sthat has been monitored since 1980 (Brockelman, 2013). The forest type is tropical seasonal evergreen, representing upland forests across Southern China, Laos, Cambodia, and much of Thailand (Figure 1, Map; Ashton, 2014; Brockelman et al., 2017). The altitude of the Mo Singto plot is 720–815 m above sea level, and average precipitation and temperature during 1994–2014 were 2,200 mm and 23°C, respectively (Brockelman et al., 2017). The Mo Singto plot has 264 species in 67 families (2011 census). Family Annonaceae ranks highest in terms of number of stems (25.5% of total), and Rubiaceae and Lauraceae in terms of numbers of species (25 and 22, respectively). The most abundant tree species were *Polyalthia khaoyaiensis* (31.5%of stems), *Cinnamomum subavenium* (9.7% of stems), and *Knema elegans* (8.4% of stems). Inventory of liana species on the Mo Singto ForestGEO Plot in Khao Yai National Park began in 2000. An individual liana is categorized as a “ramet”—the climbing stem on a host tree—or a “genet,” a stem rooted in the ground. All ramets with DBH ≥ 2 cm were measured 1.3 m vertical distance from the ground. The most abundant liana species were *Uncaria macrophylla* and *U. scandens* (Rubiaceae), common species specializing in tree-fall gaps.

### Seed Sampling and Measurement

Plant voucher specimens, including seeds, are stored in the BBH herbarium at BIOTEC of the National Science and Technology Development Agency, Thailand. Many seeds (38.3%) have been collected from gibbons’ feces during gibbon monitoring. Most seeds of species dispersed by gibbons (through defecation) have hard seed coats and do not change in their morphology during gut passage. However, most seeds (46.4%) were collected while monitoring plant phenology. Rarely, when seeds were not available in the herbarium, mostly from rare or non-fruiting species (15.3%), we used information from field-recorded and unpublished data of Mo Singto plot.

Seed mass refers to the dry weight of endosperm and embryo including the seed coat but excluding any morphological structures, such as wings, that aid dispersal (Leishman and Westoby, 1994b). Before measuring and weighing, seeds were oven-dried at 60°C for at least 3 days. After drying, we immediately measured each seed in three dimensions: length (L), the longest linear dimension; width (W), the widest orthogonal direction to L; and thickness (T), the largest orthogonal side to both the L and W, using a Vernier caliper with 2-digit micrometer precision. Finally, we weighed the same seed on a microgram balance (PB303, Mettler, Toledo). Although water loss from the drying process may shrink seed size, it does not affect our categorized dispersal modes. The categorization is justified from swallowed seeds observed in the forest, which are slightly larger than oven-dried seeds.

### Plant Functional Trait Data

#### Leaf Traits

We followed the protocol of Pérez-Harguindeguy et al. (2013) and aimed to sample at least 32 individual plants of different sizes per species. We used at least three non-damaged leaves per individual tree to measure leaf economic spectrum traits. We collected sun-exposed leaves with a long pole, whereas trained tree climbers collected liana leaves. We scanned each leaf with a commercial scanner setting for A4-paper size with the 300-PPI resolution (points per inch) and measured leaf greenness (chlorophyll content) using a portable greenness meter, SPAD-502 (Konica Minolta, Tokyo, Japan). We measured leaf thickness and toughness three times at the upper, middle, and lower parts of each leaf blade. We used a digital micro mm Vernia Caliper and the puncture test with a Penetrometer (digital force gauge DST-5 N: Imada Co., Ltd., Aichi Japan) for the thickness and toughness measurements. Finally, we dried the same leaves at 70°C for 3 days and weighed them to obtain specific leaf area (leaf area per mass unit). To find the area of each individual leaf, we automatized leaf areas of all pictures scanned using the R package “LeafArea” (Katabuchi, 2015). Overall, we measured leaves of 146 tree species and 46 liana species (Table 1).

**Table 1:** Mean and ranges of values of all traits with available data (*N*: number of species).

Group	Traits	Mean (range)			
		Trees	*N*	Lianas	*N*
Structure	Maximum height (m)	28.3 (2.9–55.1)	147	–	
	Wood specific gravity (g cm^−3^)	0.55 (0.21–0.87)	147	–	
	Leaf area (mm^3^)	370 (44–4,235)	146	39.3 (4.1–106.7)	46
Leaf economic spectrum	Leaf toughness (newton)	0.49 (0.16–1.32)	146	0.36 (0.10–1.24)	46
	Specific leaf area (cm^2^ g^−1^)	1,144 (230–10,821)	146	167 (321–74)	46
	Leaf greenness	54.2 (41.7–66.1)	146	49.3 (68.9–32.6)	46
Dispersal	Mean body size of frugivores (kg)^*^	6.28 (0.48–24.00)	156	10.24 (0.48–24.00)	37
Succession niche^**^	Pioneer, generalist, shade-tolerant, unknown	–	156	–	–

* We assigned zero for anemochory and unknown species.

** This is a categorical variable.

#### Succession Niche Traits and Structural Traits (Wood Density and Maximum Tree Size)

We assigned succession niche, wood density, and maximum plant height traits for tree species only. To assign the succession niche, we used the multinomial model of occurrence in two different habitat types (Chazdon et al., 2011), in which our two habitat types were secondary forest (the chronosequence of small secondary forest plots) and old-growth forest (on the Mo Singto plot; Brockelman et al., 2017; Chanthorn et al., 2017). The result allowed us to recognize four types of successional niche: (1) generalist, (2) secondary forest specialist, (3) old-growth specialist, and (4) too rare to be classified (density in both secondary and old-growth forests was too low; for 13, 8.2, 50, 15.1 and 13.7% of tree species, respectively). However, we also assigned “unclassified” for species with inadequate information for the previous classification analysis. Wood density values were derived mainly from the global wood density database (Chave et al., 2009). When wood densities were not available at the species level, we used genus level and family level (for 43.2 and 17.8% of species, respectively). Maximum tree heights were calculated using the DBH–height allometric equation developed based on the dataset of Southeast Asian tropical forests (Feldpausch et al., 2011). We used the BIOMASS R package to calculate tree height (Réjou-Méchain et al., 2017).

#### Dispersal Agents and Mean Body Size of Frugivores

We used the seed dispersal modes of trees from Brockelman et al. (2017) and Chanthorn et al. (2019), whereas the data of lianas were compiled from unpublished long-term observations on the plot. However, dispersal agent information is lacking for some species, and so we assigned a dispersal mode based on fruit morphology and seed ornamentation or appendages using information available in the Flora of Thailand.^2^ For example, we considered seeds or fruits with wings, hairs, or pappus as wind-dispersed (Note that this classification may be reasonable only for tropical woody plants, but not for other plants or biomes). Some species that we were unable to classify were assigned to an unknown dispersal mode group (see Supplementary Table 3). To avoid problems of high variation of body size among frugivores in each tree species, we determined the mean body size of all animal fruit consumers from the body mass data of Kitamura et al. (2002) and Khamcha et al. (2014) (Supplementary Table 1). Intuitively, given that one fruit species is equally favored by two frugivores, we assumed the frugivore with larger body size consumes more fruit than the smaller one. For example, *Prunus javanica* is consumed and dispersed mostly by primates and hornbills, whereas large-bodied pigeons consume relatively little (McConkey and Brockelman, 2011). On the other hand, elephants are an opportunistic seed disperser of *Choerospondias axillaris* despite having very large body size (Chanthorn and Brockelman, 2008). Thus, we used an average of body size to avoid this bias. Despite highly skewed data of frugivore body sizes, using mean is better than using median in this context because of the high redundancy of smaller frugivores (e.g., many more species of small birds compared). For the rest of species whereas, we assigned zero. Thus, dispersal agents became a single continuous variable.

### Data Analysis

#### Seed Size Allometry

We estimated seed volume using the geometric formula for a 3-dimensional ellipsoid (Equation 1) or a 2-dimensional spheroid (Equation 2):


(1) or V=(πLWT)/6

(2) $$V=\left(\pi \;LW^{2}\right)/6\;$$

where V represents seed volume; L , W , and T are the length, width, and thickness of a seed (mm). For the most species (*N* = 188 species), we used the ellipsoid equation, whereas the spheroid equation was used for seeds in which only length and width were measured (eight species). Because seed mass and volume were skewed, we transformed data with natural logarithm prior to analyses. We fitted the seed mass data and all four seed geometric variables (volume, length, width, and thickness) using a linear regression model. We also visualized the distinction between models for trees and lianas. We expected that wind-dispersed species should have created negative error in the residuals, in which anemochorus seeds should have lighter weight in order to gyrate while at equal volume.

#### Phylogenetic Analysis, Trait Coordination, and Trade-Offs

We conducted comparative phylogenetic analyses to account for the influence of phylogeny in the allometric analysis. We used the phylogeny compiled by Smith and Brown (2018), which is resolved completely at the family level and contains 80% of the genera of seed plants (Spermatophyta) in the world. This phylogeny provided the best overlap with our data, demonstrating the best match at the genus level (98% of the 144 genera). We used Smith and Brown’s megaphylogeny at genus level implemented in the software V. PhyloMaker (Jin and Qian, 2019) as a backbone to generate a phylogeny. Additionally, we tested for phylogenetic signal in seed size using the function “phylosignal” in the package “*picante*” (Kembel et al., 2010). Phylogenetic generalized least square method (PGLS) was used to build regression models in phylogenetic context with the “gls” function in R-package “nlme” and phylogenetic correlation structures from the R-package “*ape*” (Paradis and Schliep, 2019). We created two candidate models—with and without accounting for phylogeny (PGLS). We performed AIC-based model selection to find the best fitting model of all trait variables (Table 1). The predictor variables of tree species included six continuous traits, that is, maximum height, wood specific gravity, leaf area, leaf toughness, specific leaf area, leaf greenness, mean body size of frugivores, and the regeneration niche (a categorical variable). We separated the analysis of lianas because of their contrasting life history and lack of data on height, wood density and regeneration niche (Table 1). We tested for correlation (Pearson’s coefficient) among all predictor variables (see Supplementary Table 2). All statistical analyses were conducted using R 4.0.2 (R Core Team, 2016). The ancestral states of seed mass were reconstructed by maximum likelihood using the function ace in ape under a Brownian motion of continuous trait evolution. The observed values for the recent species were first log10-transformed, and then the reconstructed ancestral values were back-transformed to the linear scale before plotting on the phylogenetic tree.

## Results

### Allometric Equations Among Seed Size Metrics

**Figure 2:**
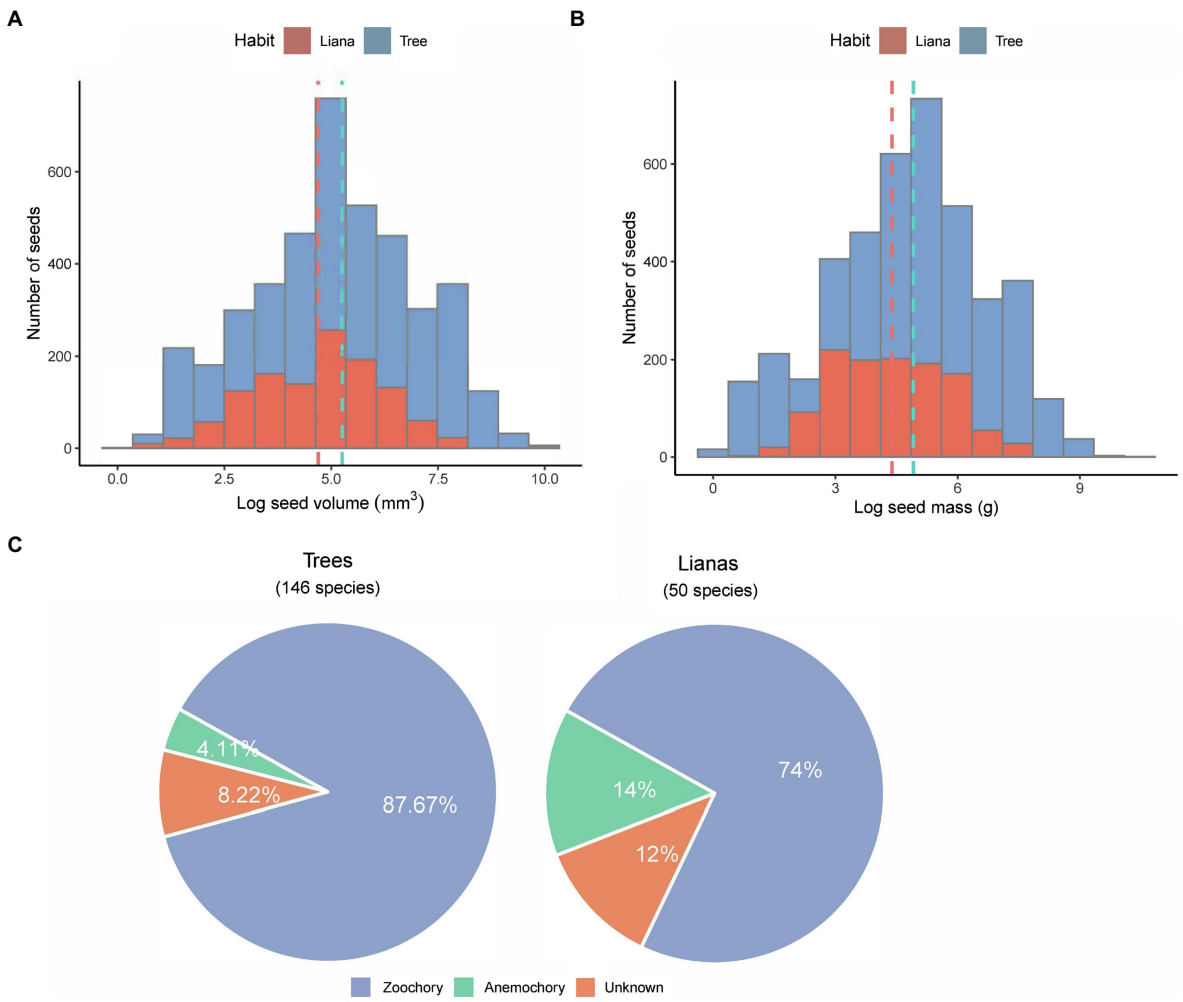
Overview of the seed size between trees and lianas. Histogram distributions of **(A)** seed volume and **(B)** seed mass. All bin widths are logarithmically scaled to facilitate comparison. **(C)** pie charts showing number of species and their dispersal modes in the study.

Across all 196 species, seed mass ranged from 0.7 mg in *Lasianthus biflorus* (Rubiaceae) to 24.5 g in *Mucuna macrocarpa* (Fabaceae). Seed volume ranged from 2.62 mm^3^ in *Diplectria barbata* (Melastomataceae) to 21,511 mm^3^ in *Mucuna macrocarpa* (Fabaceae; Supplementary Table 3). Overall, the distribution of log_e_-transformed data was symmetrical without obvious skewness in both trees and lianas (Figures 2A,B). The mean log_e_-transformed seed volume and mass of trees were larger than those of lianas [log seed volume of trees and lianas: 5.21 ± 0.03 and 4.66 ± 0.04; log seed mass of trees and lianas: 4.91 ± 0.03 and 4.39 ± 0.41 (mean ± standard error); Figures 2A,B]. Across all species, the mean seed mass of zoochorous seeds was greater (mean 0.65 ± 1.22 g) than that of anemochorus seeds (mean 0.37 ± 1.16 g). More than 87% of tree species were zoochorous (Figure 2C), whereas >74% of liana species were zoochorous (Figure 2C). Note that almost all unknown species had fleshy fruit, but we have never seen any frugivores remove the fruit, and some species had toxic fruit (e.g., *Excoecaria oppositifolia*.)

**Figure 3:**
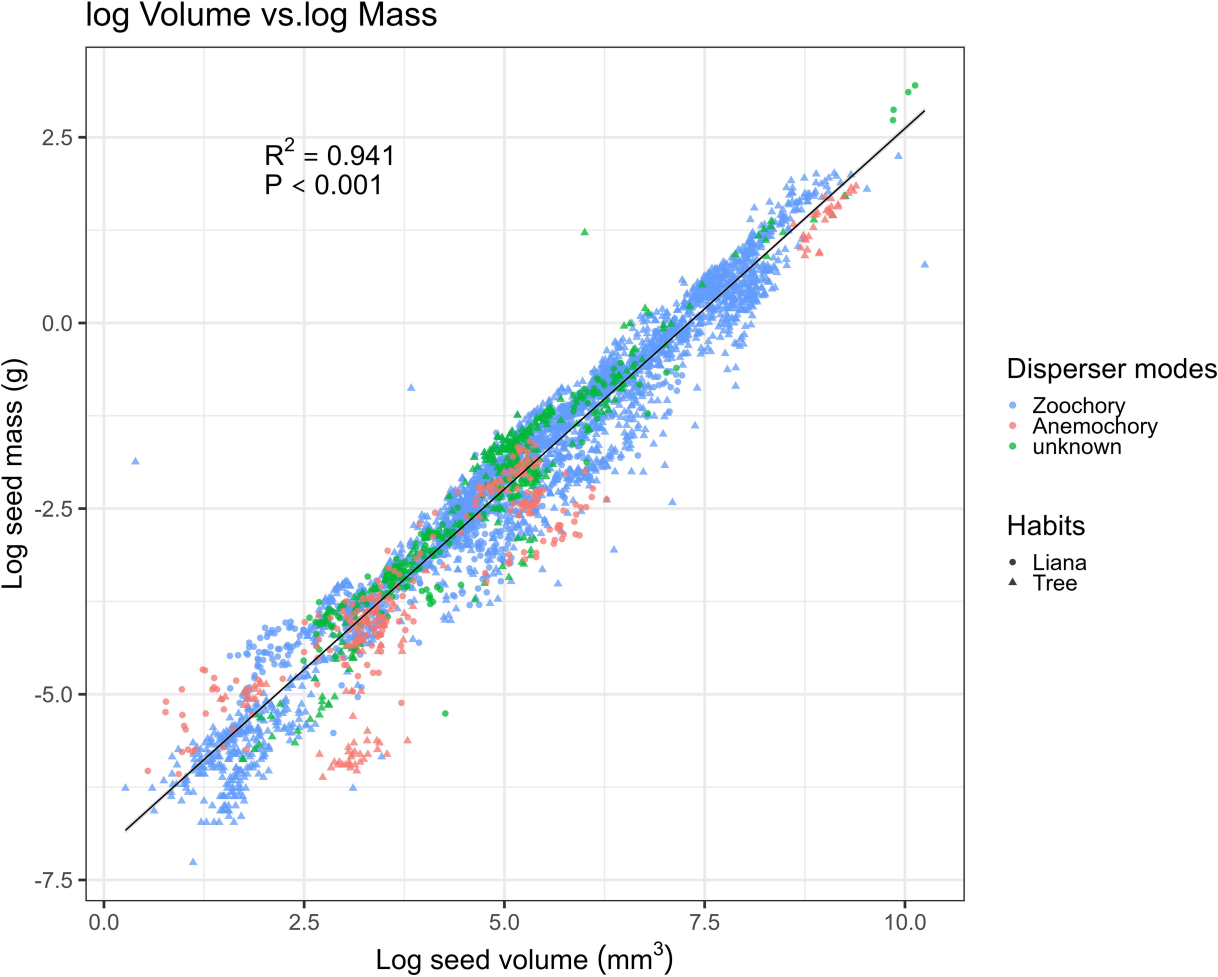
Relationship between seed mass and volume-related traits with the predicted line (black solid line) fitted by the linear regression model (*N* = 5,219). Samples are categorized into zoochory (blue), anemochory (red), and unknown (green), and tree and liana species are categorized by triangular and circular shapes.

Our fitting of the allometric equation showed the strongest positive correlations between seed volume and seed mass (*R*^2^ = 0.9414, *N* = 196; Figure 3), with random and Gaussian-distributed patterns of residuals. Thus, the equation to interchange between seed mass (g) and volume (mm^3^) was: log_e_ (seed volume) = 0.97 × log_e_(seed mass) − 7.1 (Figure 3). Surprisingly, the fitted equations with simpler geometrical metrics (Supplementary Figures 1A–C) showed the poorest fit for seed length (*R*^2^ > 0.73, *p* < 0.001; Supplementary Figure 1A), whereas the best-fit equation was with seed width (*R*^2^ > 0.87, *p* < 0.001; Supplementary Figure 1B).

### Phylogenetic Structure and Seed Size Variation

**Figure 4:**
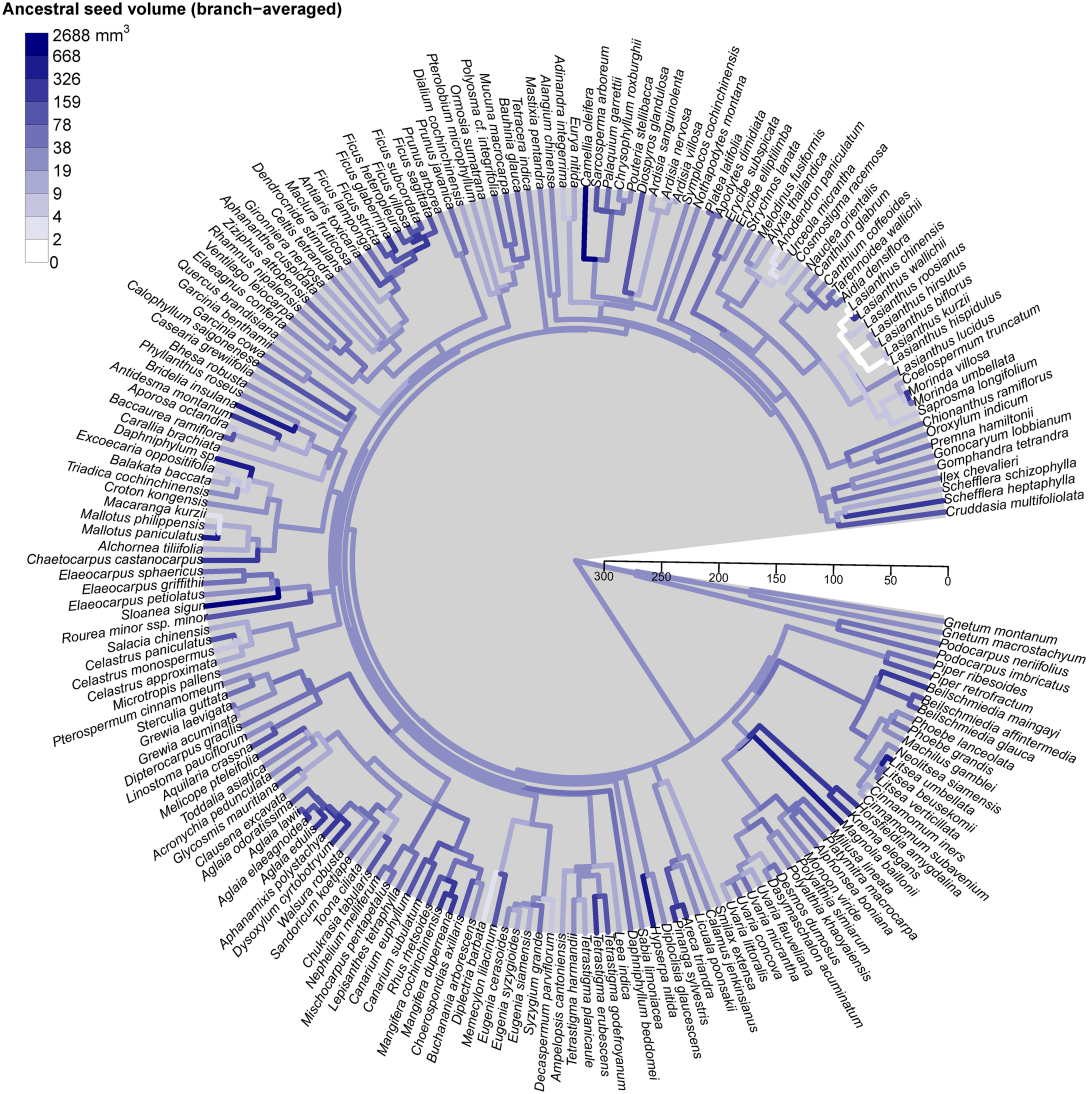
Phylogenetic tree of the 196 species found in Mo Singto plot, illustrating the evolution of mean seed size (seed volume, mm^3^). The ancestral states of this trait were reconstructed under a model of Brownian motion fitted by residual maximum likelihood (REML); the values between the two nodes of each branch were averaged to define the color as shown on the plot. The time scale units are Ma (ranging from 0 to 300 Ma).

Seed size variation was randomly distributed across the phylogenetic tree, without any specific lineages clustering with small or large seed size (Figure 4). We found no evidence of significant phylogenetic signal in different seed size measures using either mean seed volume (*K* = 0.002, *p* = 0.8), mean seed volume (*K* = 0, *p* = 0.9), or mean seed mass (*K* = 0.01, *p* = 0.7). Interestingly, most species were from different genera, suggesting that analysis at the genus-level phylogeny is reliable for ours. Note that our phylogenetic tree was constructed based on the genus level as the finest phylogenetic level. Therefore, increasing resolution at the species level is not expected to have a dramatic effect.

### Seed Trait-Plant Functional Trait Relationships

**Figure 5:**
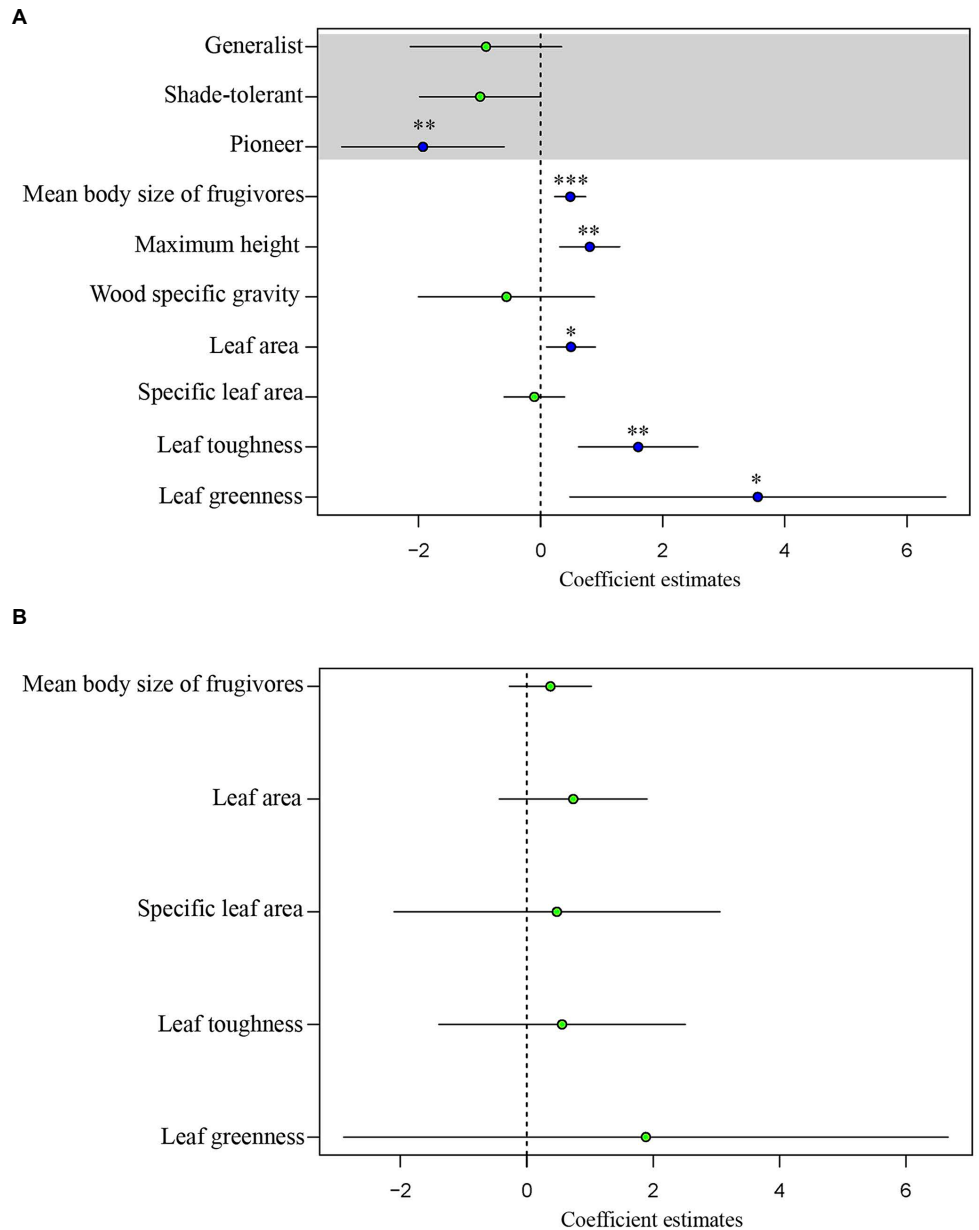
Regression coefficients estimated through generalized least squares (GLS) analysis of seed volume of **(A)** trees and **(B)** lianas. The blue dots indicate statistically significant coefficients (^*^p < 0.05, ^**^p < 0.01, ^***^p < 0.001); the green dots indicate coefficients which are non-significant (*p* ≥ 0.05). The horizontal bars show the 95% confidence intervals.

**Figure 6:**
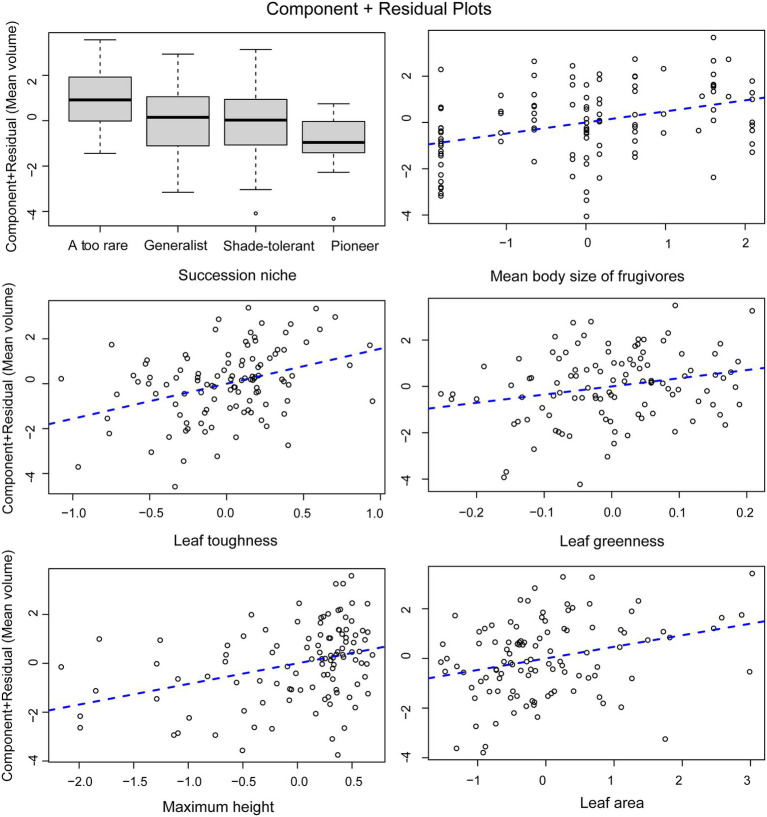
Component residual plots for the main effects in the multiple regression model. Points represent mean seed volume when all factors are held at their median values (partial residuals) and regression lines are derived from the multivariate regression model.

For tree species, the normal linear model (AIC = 411.05) provided a better fit than the PGLS model (AIC = 481.35). This result implies that phylogeny did not influence the observed patterns. For the analysis of tree species, the results of the normal linear model showed that seed size was significantly correlated with the group indicating seed dispersal (the mean body size of frugivores), three leaf traits (leaf toughness, leaf area, and leaf greenness) and the single structural trait, maximum tree height (Figure 5, Supplementary Table 4). Figure 6 shows the component residual plots of significant variables. In contrast with the liana species tree, the PGLS model fitted the seed data slightly better than the normal linear model (AIC values were 124.12 and 122.58, respectively; Supplementary Table 5). Nonetheless, there were no significant covariates.

## Discussion

### Allometry and Phylogeny

Seed size is a major trait in two large research topics: functional traits and seed dispersal (Jansen et al., 2004; Wang and Chen, 2009; Wang and Yang, 2014). However, data are generally created from different seed size metrics, for example, seed mass and geometric size (volume, length, width, thickness). Measuring two metrics of seed size together can be a laborious task, but our allometric equation helps to reduce this problem. We found high interchangeability of our models because of well-fitting log-linear models using seed mass and volume. Studies in Neotropical forests show well-fitted models similar to ours (Wright et al., 2007). Contrary to our expectation, wind dispersal did not create systematic error. However, based on our visualization, there was a cluster of highly negative residuals from the single species, *Toona ciliata*. Moreover, our best selected model was the one without any phylogenetic effects, suggesting that our allometric equations could apply in other seasonal tropical forests of Southeast Asia where our forest is well represented and shares similar phylogenetic structure (Ashton, 2014; Brockelman et al., 2017).

Species with similar seed size were not clustered in certain lineages on the phylogeny tree. They were distributed among several lineages and groups of lineages across the phylogenetic trees. In our visualization (Figure 4), we did not see any clear pattern between Gymnosperms and Angiosperms. This contrasts with the discovery of large differences between both groups in the global study of Moles et al. (2005). However, note that our Gymnosperms comprise only four species belonging to two genera, a significant limitation of our study.

### Trait–Trait Relationships: Driver and Trait Coordination

We interpret a significant positive correlation as either a driver or trait coordination, but interpret a negative correlation as a trade-off. Our results for tree species found only significant positive correlations. The strongest positive relationship was between mean body size of frugivore dispersers and seed size, without any phylogenetic effect. We interpret this trait as a major driver of seed size variation, acting as a constraint. This is a consequence of natural selection in which frugivores tend to feed on fruits with the largest seeds that they can swallow, for single-seeded fruits, such as drupes. Selection on seed size driven by frugivore gape size may occur within a few centuries (Galetti et al., 2013; Dirzo et al., 2014). In Southeast Asia, the seed size of *Choerospondias axillaris*, a primarily deer-dispersed species (Brodie et al., 2013), is smaller in the north of Thailand where deer have been extirpated for *ca.* 80 years compared to our study site, where deer are still abundant. A possible consequence is secondary extinction (Brodie et al., 2014), which may also effect ecosystem services, such as carbon sequestration (Osuri et al., 2016; Chanthorn et al., 2019). Evidence from Neotropical forests, that is, “anachronisms,” suggests that the extinction of large-sized frugivores during the Pleistocene era may have led to reduction of opportunities for long-distance dispersal in many Neotropical trees with very large-sized fruit (Janzen and Martin, 1982).

The positive correlation between nearly all traits with seed size suggests that traits may be physiologically co-adapted either with or without any mechanistic links. The positive significant relation between leaf area and chlorophyll content (indicated by leaf greenness) may be explained as co-evolved traits which support the energy demand of large seed size. Normally, a large leaf has a high photosynthetic rate, indicating a fast-growing strategy, especially in pioneer species (Poorter and Bongers, 2006; Reich, 2014). In our old-growth forest, species with large-sized leaves may have been selected to produce more energy to sustain large seeds. Although this trend is similar to observations in the global study (Díaz et al., 2016), there has been little explanation for it. Species with large leaves are typically pioneer species (Reich, 2014), which may have been selected to produce large numbers of small seeds more rapidly. In Southeast Asia, *Macaranga* spp. are examples of pioneer trees that support this hypothesis. However, leaves of dominant or common species in our secondary forests are not as large as those of *Macaranga* or *Hibiscus* (Chanthorn et al., 2017). The leaves of our pioneer species seem to be smaller and tend to have lower specific leaf area (see Supplementary Figure 2). Moreover, there was a strong negative correlation between seed size and successional niche in the secondary forest, suggesting that many pioneer species produce small seeds coordinated with small leaf size. Also, in meta-analyses of functional traits, plant size and seed size pattern correlate positively along the same principal component axis (Díaz et al., 2004, 2016). Additionally, this relates to vertical niche position in the forest canopy, or to the expectation that a canopy tree may have larger leaves to take advantage of greater exposure to the sun. We also found a positive correlation between maximum tree height and seed size. This is because the old-growth forest has taller trees, and trees that require larger seeds that have a better chance to survive in the shady understory. Although the energy demand of large seeds is supported by high leaf chlorophyll content, the exclusion of specific leaf area (SLA) among all leaf traits from the regression model suggests that larger leaf size is required by a higher photosynthetic rate rather than needed for storing substances or strengthening leaf structure. Furthermore, SLA is largely independent of leaf area (Díaz et al., 2016). Studies at regional and community scales have found that SLA is weakly related to plant height and seed size (Díaz et al., 2004) and that it often shows no relationship with wood density and leaf size across many species and community types (Wright et al., 2010). Consistent with our result that large seeds coordinate with large leaf area and high chlorophyll content, the relationship with SLA is very weak.

Interestingly, our strongest leaf economic trait was toughness (justified by the *p*-value). Our result confirms the trend found in other studies which compared forests in three continents including a tropical rainforest of Southeast Asia (Kraft et al., 2015). This trait coordination reflects the syndrome associated with a slow life-history/reproductive strategy characteristic of large-seeded species adapted to resource-limited environments, with prolonged leaf lifespans and well-defended leaves (Moles et al., 2005). These findings provide supporting evidence for correlated components of the life-history strategy, between seed size and mechanical leaf defense (Dalling et al., 2020).

However, all these relationships were absent in lianas. Leaf trait variability was generally lower in lianas than in tree species in the plot. This is likely because most mature liana species grow to the top of forest canopy, where their leaves are exposed to the same high-light conditions as tall trees (Schnitzer, 2018). Lianas are structural parasites, which germinate on the ground, stay rooted until finding other plants to use as structural support (Visser et al., 2018). Due to this growth form, lianas can acquire resources faster than trees, because they invest fewer resources in structure, and more into productive leaves and reproductive biomass (Collins et al., 2016; Schnitzer, 2018; Werden et al., 2018). For structural traits, such as height, the strong positive correlation was also similar to that found in the global study (Díaz et al., 2016). The explanation is the fecundity trade-off throughout the plants’ lifetime, in which large trees compensate low fecundity with larger seed size and high survival throughout their longer life spans (Moles, 2018). In addition, the seed volume of second-growth species was smallest, which may also be explained by the fecundity trade-off. Smaller seeded species have the fecundity advantage which allows them to disperse widely and recruit in light gaps and forest edge environments (Rose, 2005).

Wood density has been proposed as one of the most important functional traits organizing tropical forests at the population level, affecting species survival and competitive ability (Chave et al., 2009). We expected that species with large seed size would tend to have a high wood density (stem specific gravity) There is a well-supported tendency for large hardwood species to have larger fruits and seeds (Wright et al., 2007; Bello et al., 2015). However, this relationship was not significant in our study, suggesting that more studies from Southeast Asia are required to support a pattern different from that in Neotropical forests. For instance, the study by Krishnan et al. (2019) showed that tropical dry-forest trees with large seeds and low wood density had greater survival rates under drought conditions in the Indian subcontinent, which should allow them to cope with global change more efficiently. Several studies have demonstrated the impact of defaunation on carbon storage, which is based on an underlying coordination between wood density and seeds size in many tropical forests, including Asia (Osuri et al., 2016). In our forest, the wood density of trees dispersed by large-bodied frugivores tends to be higher than that of species dispersed by small birds (Chanthorn et al., 2019). However, some species with large seeds are not dispersed by large-bodied frugivores, for example, species belonging to the family Fagaceae and those with unknown dispersal mechanisms, *Excoecaria oppositifolia* (Euphorbiaceae) and *Gonocaryum lobbianum* (Cardiopteridaceae; Chanthorn et al., 2019).

## Conclusion

We created seed size allometry equations for converting between seed mass and seed volume, which is not primarily a consequence of phylogenetic effects. In addition to seed size, we compiled metadata for other functional traits and introduced a new trait, the mean body size of frugivores. The seed trait and plant functional trait relationships revealed both a driver (mean body size of frugivores) and trait coordination between seed size and other leaf traits of tree species, but there were no trade-offs (negative relationships) with other traits. This pattern was not due to any phylogenetic effect. In contrast, the results of the corresponding analysis of lianas revealed the absence of all patterns. These results do not support all findings from other or global studies (Díaz et al., 2016; Moles, 2018). Our study therefore highlights the importance of local community studies, where all species are subject to the same ecological conditions.

## Data Availability Statement

The original contributions presented in the study are included in the article/Supplementary Material, further inquiries can be directed to the corresponding authors. The data supported this study is openly available at the following URL/DOI: (https://zenodo.org/deposit/6466998).

## Author Contributions

PP and WC conceived and designed the study with input from EP. PP, WB, TK, NL, AN, and RS collected, weighed, and measured seed size. PT and WC collected liana functional traits. PP, EP, PT, and WC manipulated and analyzed the data. PP and WC wrote the first draft of manuscript. All authors read the manuscript, provided feedback, and approved the submitted version.

## Funding

This study was funded by the Postdoctoral Fellowship Program of Kasetsart University (2020–2021) and the Alexander von Humboldt Foundation and Thailand Research Fund (RSA6180050).

## Conflict of Interest

The authors declare that the research was conducted in the absence of any commercial or financial relationships that could be construed as potential conflicts of interest.

## Publisher’s Note

All claims expressed in this article are solely those of the authors and do not necessarily represent those of their affiliated organizations, or those of the publisher, the editors and the reviewers. Any product that may be evaluated in this article, or claim that may be made by its manufacturer, is not guaranteed or endorsed by the publisher.

## Footnotes

^1^ www.forestgeo.si.edu

^2^ www.dnp.go.th/botany/eflora
