# Supplementary material for: Seed Size Variation of Trees and Lianas in a Tropical Forest of Southeast Asia: Allometry, Phylogeny, and Seed Trait - Plant Functional Trait Relationships
Source: Front Plant Sci. 2022 May 17;13:852167. doi: 10.3389/fpls.2022.852167 (PMC9165448; doi:10.3389/fpls.2022.852167)
Supplement: Supplementary file 1 [file Data_Sheet_1.pdf]

Supplementary Material

Seed Size Variation of Trees and Lianas in a Tropical Forest of Southeast Asia: Allometry, Phylogeny, and Seed Trait - Plant Functional Trait Relationships

Pornwiwan Pothasin, Emmanuel Paradis, Warren Y Brockelman, Anuttara Nathalang, Thantiyapawn Khemrugka, Noppawan Lomwong, Patcharapan Thripob, Rampai Saenprasert and Wirong Chanthorn

Supplementary Table 1. Frugivores and their body sizes (weight Seed plant classification in relation to body weight

Frugivores	Weight (kg)	References
Small birds: puff-throated bulbul (smallest) and imperial pigeon (largest)	0.42-0.55	Kitamura et al. 2002, Kitamura 2011, Khamcha et al. 2014
Hornbills	0.75-2.6	Kitamura et al. 2002
Macaque & Gibbon	5.5	Kitamura et al. 2002
Terrestrial mammal: civets, deer bears and elephants.	3.5-4000	Kitamura et al. 2002

Supplementary Table 2. Pearson correlation coefficients of trait variables; structure, leaf economic spectrum (leaf toughness, specific leaf area and leaf greenness, mean body weight of frugivores and succession niche trait variables.

Correlation table for tree variables

Variable	Leaf toughness	Mean body weight of frugivores	Maximum plant height	Wood density gravity	Specific leaf area	Greenness	Leaf area
Leaf toughness		-0.026	-0.158	0.221	-0.093	0.339	0.184
Mean body weight of frugivores			0.072	-0.040	-0.013	-0.033	-0.002
Maximum plant height				-0.091	-0.092	0.010	-0.103
Wood specific gravity					0.015	0.101	0.002
Specific leaf area						0.152	0.536
Greenness							0.249
Leaf area							

Correlation table for liana variables

	Leaf toughness	Mean body weight of frugivores	Specific leaf area	Greenness	Leaf area
Leaf toughness		0.246	-0.523	0.5998	0.377
Mean body weight of frugivores			-0.339	0.219	-0.133
Specific leaf area				-0.732	-0.029
Greenness					0.068
Leaf area					

Supplementary Table 3. Mean seed mass, length, volume, width and thickness and number of seed samples of all species used in this study (More data is available at https://zenodo.org/deposit/6466998).

Note: * indicates species that used the mean genus wood density when without data at the species level from the global wood density database (Chave et al., 2009). ** lianas species were not previously reported in TRY global databases. *** indicates dispersal mode based on fruit morphology and seed ornamentation or appendages using information available in the Flora of Thailand (https://www.dnp.go.th/botany/eflora/index.html).

Abbreviations: G, Gibbons' feces; H, Herbarium; F, Field-recorded data. SG, Second growth/Secondary forest; OG, Old growth forest, Species not previously analyzed for habitat classification are shown as unclassified.

Order	Family	Species	Habit	Mean Length (mm)	Mean Width (mm)	Mean Thickness (mm)	Mean Mass (g)	*n*	Source	Regeneration niche	Dispersal mode
APIALES	Araliaceae	*Schefflera heptaphylla**	Tree	4.08	2.94	1.04	0.005	30	G	OG specialist	Zoochory
APIALES	Araliaceae	*Schefflera schizophylla**	Tree	3.68	3.23	2.01	0.008	2	G	Unclassified	Zoochory
AQUIFOLIALES	Aquifoliaceae	*Ilex chevalieri**	Tree	3.63	1.90	1.54	0.005	30	G	OG specialist	Zoochory
AQUIFOLIALES	Cardiopteridaceae	*Gonocaryum lobbianum**	Tree	40.72	21.89	19.61	4.815	4	H	OG specialist	Unknown
AQUIFOLIALES	Stemonuraceae	*Gomphandra tetrandra**	Tree	16.14	7.13	6.89	0.228	30	G	SG specialist	Zoochory
ARECALES	Arecaceae	*Areca triandra**	Tree	41.27	18.20	-	-	30	F	OG specialist	Zoochory
ARECALES	Arecaceae	*Licuala poonsakii**	Tree	7.29	5.85	5.70	-	30	F	OG specialist	Unknown
ARECALES	Arecaceae	*Pinanga sylvestris**	Tree	15.24	7.25	-	-	30	F	OG specialist	Zoochory
ARECALES	Arecaceae	*Daemonoropsis jenkisiana**	Tree	9.98	8.71	6.52	0.389	30	H	Unclassified	Unknown
CELASREALES	Celastraceae	*Microtropis pallens**	Tree	10.15	7.72	6.56	-	30	F	Too rare	Unknown
CELASREALES	Celastraceae	*Bhesa robusta**	Tree	17.82	7.92	5.66	0.149	47	H	Too rare	Zoochory
CORNALES	Cornaceae	*Alangium chinense*	Tree	8.28	6.27	4.53	0.121	11	H	Too rare	Zoochory
CORNALES	Nyssaceae	*Mastixia pentandra*	Tree	18.96	12.77	11.21	1.009	58	H	OG specialist	Zoochory
ERICALES	Ebenaceae	*Diospyros glandulosa**	Tree	14.87	9.92	2.36	0.186	76	G	SG specialist	Zoochory
ERICALES	Pentaphylacaceae	*Adinandra integerrima*	Tree	2.63	2.08	1.05	0.003	30	H	SG specialist	Zoochory
ERICALES	Pentaphylacaceae	*Eurya nitida*	Tree	15.03	11.27	4.27	-	30	F	SG specialist	Zoochory
ERICALES	Primulaceae	*Ardisia sanguinolenta**	Tree	4.86	4.61	4.47	0.040	60	G	OG specialist	Zoochory
ERICALES	Primulaceae	*Ardisia nervosa**	Tree	4.79	5.76	-	-	30	F	OG specialist	Zoochory

Order	Family	Species	Habit	Mean Length (mm)	Mean Width (mm)	Mean Thickness (mm)	Mean Mass (g)	*n*	Source	Regeneration niche	Dispersal mode
ERICALES	Primulaceae	*Ardisia villosa**	Tree	6.97	6.90	6.80	0.132	2	H	Unclassified	Zoochory
ERICALES	Sapotaceae	*Palaquium garrettii**	Tree	25.73	14.33	11.96	1.504	30	G	OG specialist	Zoochory
ERICALES	Sapotaceae	*Pouteria stellibacca**	Tree	15.81	9.15	5.90	0.386	30	G	Too rare	Zoochory
ERICALES	Sapotaceae	*Sarcosperma arboreum*	Tree	16.37	9.05	8.05	0.182	49	H	OG specialist	Zoochory
ERICALES	Sapotaceae	*Donella lanceolata**	Tree	14.36	8.04	5.05	0.228	3	H	Unclassified	Zoochory
ERICALES	Symplocaceae	*Symplocos cochinchinensis*	Tree	5.41	4.66	5.12	0.076	47	G	Generalist	Zoochory
ERICALES	Theaceae	*Camellia oleifera**	Tree	15.27	12.74	8.38	0.409	30	H	OG specialist	Zoochory
ESCALLONIALES	Polyosmaceae	*Polyosma cf. integrifolia*	Tree	7.85	6.82	6.79	0.110	52	G	OG specialist	Zoochory
FABALES	Fabaceae-Caesalpiniodeae	*Dialium cochinchinensis**	Tree	8.81	7.05	4.01	0.161	14	H	Unclassified	Unknown
FABALES	Fabaceae-Papilionoideae	*Ormosia sumatrana*	Tree	9.10	8.90	6.03	0.334	30	H	Generalist	Zoochory
FABALES	Fabaceae-Papilionoideae	*Cruddasia multifoliolate**	Tree	5.71	5.21	1.31	0.018	1	H	Unclassified	Unknown
FABALES	Fagaceae	*Quercus brandisiana*	Tree	20.19	16.93	16.21	1.534	7	H	Unclassified	Zoochory
GENTIANALES	Bignoniaceae	*Oroxylum indicum**	Tree	34.57	20.21	0.99	0.134	2	H	Too rare	Zoochory
GENTIANALES	Rubiaceae	*Nauclea orientalis*	Tree	9.77	7.40	1.00	-	30	F	OG specialist	Zoochory
GENTIANALES	Rubiaceae	*Tarennoidea wallichii**	Tree	17.50	13.39	6.97	0.701	9	H	Generalist	Zoochory
GENTIANALES	Rubiaceae	*Lasianthus kurzii**	Tree	3.56	2.09	1.61	0.003	30	H	OG specialist	Zoochory
GENTIANALES	Rubiaceae	*Saprosma longifolium**	Tree	5.79	3.12	2.89	0.021	30	H	OG specialist	Zoochory
GENTIANALES	Rubiaceae	*Canthium coffeoides**	Tree	15.52	9.11	8.19	0.448	30	G	Generalist	Zoochory
GENTIANALES	Rubiaceae	*Lasianthus lucidus**	Tree	4.51	2.53	2.03	-	30	F	OG specialist	Zoochory
GENTIANALES	Rubiaceae	*Lasianthus hirsutus**	Tree	4.86	2.83	2.43	0.009	21	H	OG specialist	Zoochory
GENTIANALES	Rubiaceae	*Lasianthus wallichii**	Tree	4.43	2.31	1.90	0.005	30	H	OG specialist	Zoochory
GENTIANALES	Rubiaceae	*Lasianthus hispidulus**	Tree	3.90	1.84	1.88	0.004	9	H	OG specialist	Zoochory
GENTIANALES	Rubiaceae	*Lasianthus roosianus**	Tree	3.65	1.87	1.44	0.002	30	H	OG specialist	Zoochory
GENTIANALES	Rubiaceae	*Lasianthus chinensis**	Tree	3.21	1.84	1.55	0.002	30	H	OG specialist	Zoochory
GENTIANALES	Rubiaceae	*Lasianthus biflorus**	Tree	2.55	1.90	1.79	0.004	30	H	Unclassified	Zoochory
GENTIANALES	Rubiaceae	*Canthium glabrum*	Tree	10.07	5.75	4.64	-	30	F	OG specialist	Zoochory
GENTIANALES	Rubiaceae	*Aidia densiflora**	Tree	16.53	10.90	4.33	-	30	F	OG specialist	Zoochory

Order	Family	Species	Habit	Mean Length (mm)	Mean Width (mm)	Mean Thickness (mm)	Mean Mass (g)	*n*	Source	Regeneration niche	Dispersal mode
ICACINALES	Icacinaceae	*Nothapodytes montana**	Tree	19.60	14.28	12.46	1.077	10	H	Generalist	Zoochory
ICACINALES	Icacinaceae	*Platea latifolia*	Tree	31.07	14.19	13.95	1.170	15	G	OG specialist	Zoochory
ICACINALES	Icacinaceae	*Apodytes dimidiata*	Tree	8.10	5.69	3.46	0.071	30	H	Generalist	Zoochory
LAMIALES	Oleaceae	*Chionanthus ramiflorus*	Tree	19.31	10.02	9.81	0.798	10	G	OG specialist	Zoochory
LAURALES	Lauraceae	*Litsea beusekomii**	Tree	35.06	20.93	18.46	5.921	9	H	OG specialist	Zoochory***
LAURALES	Lauraceae	*Litsea umbellata*	Tree	6.22	4.99	4.82	0.077	30	H	OG specialist	Zoochory
LAURALES	Lauraceae	*Litsea verticillate**	Tree	8.15	6.03	5.24	-	30	F	OG specialist	Zoochory***
LAURALES	Lauraceae	*Machilus gamblei*	Tree	6.17	7.56	7.61	-	30	F	OG specialist	Zoochory
LAURALES	Lauraceae	*Phoebe lanceolata*	Tree	9.87	5.83	-	-	30	F	OG specialist	Zoochory
LAURALES	Lauraceae	*Neolitsea siamensis**	Tree	5.55	5.59	5.30	-	23	F	OG specialist	Zoochory
LAURALES	Lauraceae	*Phoebe grandis*	Tree	15.96	10.76	10.51	0.811	7	H	Too rare	Zoochory
LAURALES	Lauraceae	*Beilschmiedia affintermedia**	Tree	26.12	12.83	12.25	1.566	15	H	OG specialist	Zoochory
LAURALES	Lauraceae	*Beilschmiedia glauca**	Tree	18.02	9.54	9.43	0.577	30	H	OG specialist	Zoochory
LAURALES	Lauraceae	*Beilschmiedia maingayi**	Tree	14.11	10.75	10.18	0.361	15	G	OG specialist	Zoochory
LAURALES	Lauraceae	*Cinnamomum iners*	Tree	9.18	5.63	5.18	0.103	3	H	OG specialist	Zoochory
LAURALES	Lauraceae	*Cinnamomum subavenium*	Tree	12.25	7.66	6.93	0.309	30	G	OG specialist	Zoochory
MAGNOLIALES	Annonaceae	*Alphonsea boniana**	Tree	13.98	9.90	5.34	0.354	30	G	OG specialist	Zoochory
MAGNOLIALES	Annonaceae	*Miliusa lineata**	Tree	11.86	8.55	8.15	0.266	3	G	OG specialist	Zoochory
MAGNOLIALES	Annonaceae	*Platymitra macrocarpa*	Tree	29.47	17.36	11.33	1.462	139	G	OG specialist	Zoochory
MAGNOLIALES	Annonaceae	*Polyalthia khaoyaiensis**	Tree	7.22	7.02	6.30	0.200	62	G	OG specialist	Zoochory
MAGNOLIALES	Annonaceae	*Polyalthia simiarum*	Tree	23.27	13.88	13.22	1.579	6	G	OG specialist	Zoochory
MAGNOLIALES	Annonaceae	*Monoon viridis**	Tree	25.61	14.57	13.96	2.311	11	G	Unclassified	Zoochory
MAGNOLIALES	Annonaceae	*Dasymaschalon acuminatum**	Tree	14.38	4.37	4.09	0.143	30	H	OG specialist	Zoochory
MAGNOLIALES	Magnoliaceae	*Magnolia baillonii**	Tree	6.87	5.05	2.87	0.052	60	H	Generalist	Zoochory
MAGNOLIALES	Myristicaceae	*Horsfieldia amygdalina*	Tree	23.33	20.23	19.37	-	30	F	Generalist	Zoochory
MAGNOLIALES	Myristicaceae	*Knema elegans**	Tree	19.23	13.91	13.44	1.739	30	G	OG specialist	Zoochory
MALPIGHIALES	Callophyllaceae	*Calophyllum saigonenese**	Tree	8.06	6.66	6.49	0.102	30	H	Too rare	Unknown
MALPIGHIALES	Clusiaceae	*Garcinia benthamii**	Tree	21.07	13.50	8.84	-	30	F	OG specialist	Zoochory

Order	Family	Species	Habit	Mean Length (mm)	Mean Width (mm)	Mean Thickness (mm)	Mean Mass (g)	*n*	Source	Regeneration niche	Dispersal mode
MALPIGHIALES	Clusiaceae	*Garcinia cowa**	Tree	19.08	10.07	6.22	0.392	10	H	Unclassified	Zoochory***
MALPIGHIALES	Euhorbiaceae	*Alchornea tiliifolia**	Tree	7.61	4.92	3.68	0.033	13	H	OG specialist	Zoochory
MALPIGHIALES	Euhorbiaceae	*Mallotus paniculatus*	Tree	3.21	3.08	2.77	0.016	30	H	Too rare	Zoochory
MALPIGHIALES	Euhorbiaceae	*Mallotus philippensis*	Tree	4.02	3.90	3.59	0.017	14	H	Too rare	Zoochory
MALPIGHIALES	Euhorbiaceae	*Triadica cochinchinensis**	Tree	4.10	3.58	2.96	0.012	3	H	Too rare	Unknown
MALPIGHIALES	Euhorbiaceae	*Aporosa octandra**	Tree	7.25	5.38	3.28	0.045	35	H	OG specialist	Zoochory
MALPIGHIALES	Euhorbiaceae	*Balakata baccata**	Tree	6.96	4.86	4.58	0.055	30	G	Generalist	Zoochory
MALPIGHIALES	Euhorbiaceae	*Chaetocarpus castanocarpus*	Tree	14.38	12.03	10.20	0.175	8	H	SG specialist	Zoochory
MALPIGHIALES	Euhorbiaceae	*Excoecaria oppositifolia**	Tree	3.59	3.55	-	-	30	F	OG specialist	Unknown
MALPIGHIALES	Euhorbiaceae	*Macaranga kurzii**	Tree	4.21	4.14	3.74	0.035	12	H	Unclassified	Zoochory
MALPIGHIALES	Euhorbiaceae	*Phyllanthus roseus**	Tree	6.55	3.55	3.27	0.027	4	H	Unclassified	Zoochory
MALPIGHIALES	Euhorbiaceae	*Croton kongensis**	Tree	4.24	3.52	3.23	0.025	13	H	Unclassified	Unknown
MALPIGHIALES	Phyllanthaceae	*Antidesma montanum*	Tree	4.81	3.56	2.48	-	30	F	OG specialist	Zoochory
MALPIGHIALES	Phyllanthaceae	*Baccaurea ramiflora**	Tree	11.65	8.75	3.20	0.062	30	G	OG specialist	Zoochory
MALPIGHIALES	Phyllanthaceae	*Bridelia insulana*	Tree	8.60	5.25	4.86	0.066	30	G	OG specialist	Zoochory
MALPIGHIALES	Rhizophoraceae	*Carallia brachiata*	Tree	5.89	5.25	4.90	-	50	F	SG specialist	Zoochory
MALPIGHIALES	Salicaceae	*Casearia grewiifolia*	Tree	3.87	2.94	1.80	0.006	27	H	OG specialist	Zoochory
MALVALES	Dipterocarpaceae	*Dipterocarpus gracilis*	Tree	29.55	23.09	22.69	4.229	30	H	OG specialist	Anemochory***
MALVALES	Malvaceae	*Pterospermum cinnamomeum**	Tree	9.92	6.61	2.92	0.091	30	H	Generalist	Anemochory***
MALVALES	Sterculiaceae	*Sterculia guttata**	Tree	14.73	9.04	8.23	0.544	19	H	Too rare	Zoochory
MALVALES	Thymelaeaceae	*Aquilaria crassna**	Tree	15.15	6.88	5.96	0.115	30	G	OG specialist	Zoochory
MALVALES	Tiliaceae	*Grewia laevigata**	Tree	8.01	7.45	5.14	0.157	30	H	Unclassified	Unknown
MYRTALES	Melastomataceae	*Memecylon lilacinum*	Tree	7.16	6.11	5.29	0.123	21	G	Generalist	Zoochory
MYRTALES	Myrtaceae	*Eugenia siamensis**	Tree	24.72	22.02	21.37	4.576	2	G	OG specialist	Zoochory
MYRTALES	Myrtaceae	*Eugenia syzygioides*	Tree	7.69	6.11	5.22	0.110	11	G	SG specialist	Zoochory
MYRTALES	Myrtaceae	*Syzygium nervosum*	Tree	7.97	6.36	5.52	0.121	22	G	SG specialist	Zoochory
MYRTALES	Myrtaceae	*Decaspermum parviflorum**	Tree	3.16	2.40	1.31	-	30	F	Generalist	Zoochory
MYRTALES	Myrtaceae	*Syzygium grande**	Tree	9.91	8.15	4.99	0.193	5	G	Unclassified	Zoochory

Order	Family	Species	Habit	Mean Length (mm)	Mean Width (mm)	Mean Thickness (mm)	Mean Mass (g)	*n*	Source	Regeneration niche	Dispersal mode
OXALIDALES	Elaeocarpaceae	*Sloanea sigun*	Tree	9.20	4.86	4.53	0.108	41	H	OG specialist	Zoochory
OXALIDALES	Elaeocarpaceae	*Elaeocarpus griffithii**	Tree	14.44	6.72	6.61	0.207	44	G	Too rare	Zoochory
OXALIDALES	Elaeocarpaceae	*Elaeocarpus petiolatus*	Tree	18.52	17.07	16.00	2.919	10	G	Too rare	Zoochory
OXALIDALES	Elaeocarpaceae	*Elaeocarpus sphaericus*	Tree	19.64	18.86	17.99	2.769	29	G	OG specialist	Zoochory
PINALES	Podocarpaceae	*Podocarpus imbricatus**	Tree	6.25	5.20	5.01	0.071	30	H	Too rare	Zoochory
PINALES	Podocarpaceae	*Podocarpus neriifolius*	Tree	8.28	6.63	6.27	0.176	8	G	OG specialist	Zoochory
RHAMNALES	Lauraceae	*Leea indica*	Tree	4.45	3.48	2.94	0.022	30	H	OG specialist	Zoochory
ROSALES	Cannabaceae	*Gironniera nervosa*	Tree	5.11	4.29	3.22	0.047	30	G	OG specialist	Zoochory
ROSALES	Cannabaceae	*Celtis tetrandra*	Tree	6.74	5.37	5.14	0.111	30	G	Too rare	Zoochory
ROSALES	Moraceae	*Antiaris toxicaria*	Tree	19.34	15.21	13.34	1.201	6	G	OG specialist	Zoochory
ROSALES	Moraceae	*Ficus subcordata**	Tree	27.27	19.82	20.35	4.899	30	G	Too rare	Zoochory
ROSALES	Moraceae	*Ficus stricta**	Tree	15.90	14.86	20.39	1.515	30	G	Too rare	Zoochory
ROSALES	Moraceae	*Ficus lamponga**	Tree	13.65	14.00	13.79	0.882	30	H	Too rare	Zoochory
ROSALES	Moraceae	*Ficus glaberrima**	Tree	10.39	9.70	10.31	0.453	30	G	Too rare	Zoochory
ROSALES	Moraceae	*Ficus heteropleura**	Tree	10.03	9.60	9.72	0.342	60	H	Unclassified	Zoochory
ROSALES	Rosaceae	*Prunus arborea*	Tree	8.02	6.55	5.56	0.152	30	G	Too rare	Zoochory
ROSALES	Rosaceae	*Prunus javanica*	Tree	15.56	9.20	8.75	0.486	28	G	OG specialist	Zoochory
ROSALES	Urticaceae	*Dendrocnide stimulans**	Tree	3.62	2.93	0.84	0.004	30	H	OG specialist	Zoochory
SAPINDALES	Anacardiaceae	*Mangifera cochinchinensis**	Tree	35.48	17.36	14.36	3.967	6	H	OG specialist	Zoochory
SAPINDALES	Anacardiaceae	*Mangifera duperreana*	Tree	48.47	27.04	21.54	7.609	3	H	OG specialist	Zoochory
SAPINDALES	Anacardiaceae	*Rhus rhetsoides**	Tree	4.57	3.58	2.09	0.026	30	H	Generalist	Zoochory
SAPINDALES	Anacardiaceae	*Buchanania arborescens*	Tree	9.08	6.59	4.41	0.093	29	G	SG specialist	Zoochory
SAPINDALES	Anacardiaceae	*Choerospondias axillaris*	Tree	18.29	13.75	13.34	1.676	9	G	Generalist	Zoochory
SAPINDALES	Burseraceae	*Canarium euphyllum*	Tree	31.98	14.59	13.79	3.029	50	H	Generalist	Zoochory
SAPINDALES	Burseraceae	*Canarium subulatum**	Tree	39.54	15.32	14.78	4.402	2	H	Unclassified	Zoochory***
SAPINDALES	Meliaceae	*Sandoricum koetjape*	Tree	27.04	14.97	11.44	1.390	30	G	OG specialist	Zoochory
SAPINDALES	Meliaceae	*Aphanamixis polystachya*	Tree	15.06	13.71	11.33	1.099	16	H	OG specialist	Zoochory
SAPINDALES	Meliaceae	*Toona ciliata*	Tree	16.72	4.39	0.61	0.003	30	H	Too rare	Anemochory

Order	Family	Species	Habit	Mean Length (mm)	Mean Width (mm)	Mean Thickness (mm)	Mean Mass (g)	*n*	Source	Regeneration niche	Dispersal mode
SAPINDALES	Meliaceae	*Walsura robusta*	Tree	12.09	10.45	9.83	-	30	F	OG specialist	Zoochory
SAPINDALES	Meliaceae	*Aglaia edulis*	Tree	15.79	9.81	7.69	0.762	29	G	Generalist	Zoochory
SAPINDALES	Meliaceae	*Aglaia elaeagnoidea*	Tree	10.15	8.13	4.55	0.240	30	G	OG specialist	Zoochory
SAPINDALES	Meliaceae	*Aglaia lawii*	Tree	29.80	18.66	13.92	4.720	11	H	OG specialist	Zoochory
SAPINDALES	Meliaceae	*Chukrasia tabularis**	Tree	9.35	5.98	1.02	0.016	30	H	Too rare	Anemochory
SAPINDALES	Meliaceae	*Dysoxylum cyrtobotryum**	Tree	14.66	11.58	8.89	1.018	5	H	OG specialist	Zoochory
SAPINDALES	Meliaceae	*Aglaia odoratissima**	Tree	12.29	10.19	8.51	0.628	14	G	Unclassified	Zoochory
SAPINDALES	Rutaceae	*Glycosmis mauritiana**	Tree	4.99	6.45	5.61	-	30	F	OG specialist	Zoochory
SAPINDALES	Rutaceae	*Melicope pteleifolia**	Tree	2.95	2.21	1.81	0.007	15	H	Generalist	Anemochory
SAPINDALES	Rutaceae	*Clausena excavate**	Tree	7.28	5.11	3.76	0.084	30	H	SG specialist	Zoochory
SAPINDALES	Rutaceae	*Acronychia pedunculata**	Tree	7.94	5.67	5.12	0.080	3	H	Unclassified	Zoochory
SAPINDALES	Sapindaceae	*Mischocarpus pentapetalus**	Tree	8.30	7.48	7.04	0.182	24	H	OG specialist	Zoochory
SAPINDALES	Sapindaceae	*Nephelium melliferum**	Tree	23.44	14.20	10.05	1.600	30	G	OG specialist	Zoochory
SAPINDALES	Sapindaceae	*Lepisanthes tetraphylla*	Tree	16.01	8.45	7.93	0.391	27	H	Generalist	Unknown
SAPINDALES	Ulmaceae	*Aphananthe cuspidata*	Tree	10.23	8.54	6.81	-	35	F	OG specialist	Zoochory
SAXIFRAGALES	Daphniphyllaceae	*Daphniphyllum beddomei**	Tree	9.80	5.01	-	-	60	F	Generalist	Zoochory
SAXIFRAGALES	Daphniphyllaceae	*Daphniphylum sp.**	Tree	5.07	2.52	1.49	0.010	1	H	Unclassified	Zoochory***
CELASREALES	Celastraceae	*Salacia chinensis*	Liana	19.62	13.66	8.68	-	53	F	Unclassified	Zoochory
CELASREALES	Celastraceae	*Celastrus paniculatus*	Liana	7.21	3.93	3.09	0.032	16	G	Unclassified	Zoochory
CELASREALES	Celastraceae	*Celastrus approximata*	Liana**	5.89	1.68	1.42	0.013	30	G	Unclassified	Zoochory
CELASREALES	Celastraceae	*Celastrus monospermus*	Liana	6.83	2.92	3.04	-	30	F	Unclassified	Zoochory
DILLENIALES	Dilleniaceae	*Tetracera indica*	Liana**	5.29	4.99	3.75	0.058	3	H	Unclassified	Zoochory
FABALES	Fabaceae-Caesalpiniodeae	*Pterolobium microphyllum*	Liana**	13.85	10.35	2.72	0.090	30	H	Unclassified	Anemochory
FABALES	Fabaceae-Caesalpiniodeae	*Bauhinia glauca*	Liana**	7.87	4.22	1.95	0.029	30	H	Unclassified	Anemochory***
FABALES	Fabaceae-Papilionoideae	*Mucuna macrocarpa*	Liana	42.78	44.36	21.56	19.970	4	H	Unclassified	Unknown
GENTIANALES	Apocynaceae	*Cosmostigma racemosa*	Liana**	13.64	6.85	0.60	0.014	30	H	Unclassified	Anemochory***
GENTIANALES	Apocynaceae	*Melodinus fusiformis*	Liana**	11.12	7.84	3.44	0.094	60	G	Unclassified	Zoochory

Order	Family	Species	Habit	Mean Length (mm)	Mean Width (mm)	Mean Thickness (mm)	Mean Mass (g)	*n*	Source	Regeneration niche	Dispersal mode
GENTIANALES	Apocynaceae	*Urceola micrantha*	Liana	13.60	2.66	1.27	0.020	30	H	Unclassified	Anemochory***
GENTIANALES	Apocynaceae	*Anodendron paniculatum*	Liana	9.37	2.68	0.30	0.006	30	H	Unclassified	Anemochory***
GENTIANALES	Apocynaceae	*Alyxia thailandica*	Liana**	9.85	6.27	5.56	0.077	3	H	Unclassified	Zoochory
GENTIANALES	Loganiaceae	*Strychnos lanata*	Liana**	13.71	10.19	4.22	0.277	30	G	Unclassified	Zoochory
GENTIANALES	Rubiaceae	*Morinda umbellata*	Liana**	5.36	3.96	2.43	0.020	30	H	Unclassified	Zoochory
GENTIANALES	Rubiaceae	*Caelospermum truncatum*	Liana**	9.79	6.56	2.61	0.055	30	G	Unclassified	Zoochory
GENTIANALES	Rubiaceae	*Morinda villosa*	Liana**	6.64	4.23	2.72	0.032	30	H	Unclassified	Unknown
GNETALES	Gnetaceae	*Gnetum macrostachyum*	Liana**	14.90	8.65	8.41	0.469	30	G	Unclassified	Zoochory
GNETALES	Gnetaceae	*Gnetum montanum*	Liana	17.77	10.07	9.94	0.716	30	G	Unclassified	Zoochory
LAMIALES	Verbenaceae	*Premna hamiltonii*	Liana**	5.43	5.11	4.52	0.051	30	H	Unclassified	Unknown
LILIALES	Smilacaceae	*Smilax extensa*	Liana**	3.41	2.91	1.71	0.011	30	H	Unclassified	Zoochory
MAGNOLIALES	Annonaceae	*Uvaria fauveliana*	Liana**	12.48	7.42	4.14	0.205	20	G	Unclassified	Zoochory
MAGNOLIALES	Annonaceae	*Desmos dumosus*	Liana**	7.00	4.93	4.73	0.081	30	G	Unclassified	Zoochory
MAGNOLIALES	Annonaceae	*Uvaria littoralis*	Liana**	8.60	7.55	4.98	0.168	2	G	Unclassified	Zoochory
MAGNOLIALES	Annonaceae	*Uvaria micrantha*	Liana**	7.36	4.94	3.28	0.048	30	G	Unclassified	Zoochory
MAGNOLIALES	Annonaceae	*Uvaria concova*	Liana**	11.82	9.47	5.13	0.265	60	G	Unclassified	Zoochory
MALVALES	Thymelaeaceae	*Linostoma pauciflorum*	Liana**	9.43	7.83	7.23	0.062	30	H	Unclassified	Anemochory***
MALVALES	Tiliaceae	*Grewia acuminata*	Liana**	7.70	7.50	4.86	-	30	F	Unclassified	Unknown
MYRTALES	Melastomataceae	*Diplectria barbata*	Liana**	5.00	1.00	-	-	30	F	Unclassified	Zoochory
OXALIDALES	Connaraceae	*Rourea minor ssp. minor*	Liana**	13.09	7.75	5.86	0.305	20	G	Unclassified	Zoochory
PIPERALES	Piperaceae	*Piper retrofractum*	Liana**	5.32	3.42	-	-	30	F	Unclassified	Unknown
PIPERALES	Piperaceae	*Piper ribesoides*	Liana**	5.05	4.60	4.30	0.036	50	H	Unclassified	Zoochory
PROTEALES	Sabiaceae	*Sabia limoniacea*	Liana**	9.64	8.84	3.60	0.062	48	G	Unclassified	Zoochory
RANUNCULALES	Menispermaceae	*Diploclisia glaucescens*	Liana	14.04	8.60	5.00	0.165	29	G	Unclassified	Zoochory
RANUNCULALES	Menispermaceae	*Hypserpa nitida*	Liana	7.24	6.95	4.88	0.092	30	G	Unclassified	Zoochory
ROSACEAE	Elaeagnaceae	*Elaeagnus conferta*	Liana	27.41	8.32	7.87	0.368	19	G	Unclassified	Zoochory
ROSALES	Moraceae	*Ficus villosa*	Liana**	12.49	9.05	9.09	0.433	30	H	Unclassified	Zoochory
ROSALES	Moraceae	*Ficus sagittata*	Liana**	13.88	16.78	16.79	1.659	30	H	Unclassified	Zoochory

Order	Family	Species	Habit	Mean Length (mm)	Mean Width (mm)	Mean Thickness (mm)	Mean Mass (g)	*n*	Source	Regeneration niche	Dispersal mode
ROSALES	Moraceae	*Maclura fruticosa*	Liana**	5.52	4.29	2.59	0.029	30	G	Unclassified	Zoochory
ROSALES	Rhamnaceae	*Rhamnus nipalensis*	Liana**	14.63	11.46	8.30	0.532	3	H	Unclassified	Unknown
ROSALES	Rhamnaceae	*Ziziphus attopensis*	Liana	15.72	11.40	8.10	0.567	30	G	Unclassified	Zoochory
ROSALES	Rhamnaceae	*Ventilago leiocarpa*	Liana**	7.78	7.01	6.41	0.147	30	H	Unclassified	Anemochory***
SAPINDALES	Rutaceae	*Toddalia asiatica*	Liana	5.11	3.44	2.07	0.021	60	G	Unclassified	Zoochory
SOLANALES	Convolvulaceae	*Erycibe subspicata*	Liana**	12.54	10.51	9.77	0.417	5	G	Unclassified	Zoochory
SOLANALES	Convolvulaceae	*Erycibe elliptilimba*	Liana**	17.19	9.39	6.63	0.515	30	G	Unclassified	Zoochory
VITALES	Vitaceae	*Tetrastigma harmandii*	Liana**	11.79	7.10	5.66	-	30	F	Unclassified	Zoochory
VITALES	Vitaceae	*Ampelopsis cantoniensis*	Liana**	4.56	3.80	2.64	0.014	30	G	Unclassified	Zoochory
VITALES	Vitaceae	*Tetrastigma godefroyanum*	Liana**	8.37	6.55	6.15	0.173	30	G	Unclassified	Zoochory
VITALES	Vitaceae	*Tetrastigma erubescens*	Liana**	11.91	6.79	4.79	0.095	8	H	Unclassified	Zoochory
VITALES	Vitaceae	*Tetrastigma planicaule*	Liana**	11.64	6.64	5.27	0.198	30	G	Unclassified	Zoochory

Supplementary Table 4. Results of GLS and PGLS models with the coefficient, standard error, t-value, and p-value for each trait. p < 0.05, * p < 0.01, ** p < 0.001, ***. Bold character indicates traits significantly related to seed volume of tree species.

	Coefficient	Std.Error	t-value	p-value
**GLS models**				
(Intercept)	**8.564**	**0.469**	**18.275**	**0.0000*** **
Succession Type (Generalist)	-0.895	0.632	-1.415	0.1604
Succession Type (OG Specialist)	-0.991	0.507	-1.955	0.0535
Succession Type (SG Specialist)	**-1.928**	**0.679**	**-2.840**	**0.0055** **
Mean body wight of seed disperser	**0.486**	**0.129**	**3.779**	**0.0003*** **
Leaf toughness	**1.598**	**0.498**	**3.209**	**0.0018** **
Wood density gravity	-0.559	0.735	-0.761	0.4483
Specific leaf area	-0.102	0.253	-0.405	0.6867
Leaf greenness	3.557	1.570	2.266	0.0257*
Maximum plant height	**0.806**	**0.250**	**3.223**	**0.0017** **
Leaf area	**0.497**	**0.204**	**2.444**	**0.0164***
**PGLS models**				
(Intercept)	**7.222**	**3.355**	**2.153**	**0.0338** **
Succession Type (Generalist)	1.4653	0.895	-1.639	0.1049
Succession Type (OG Specialist)	-0.291	0.760	-0.383	0.7022
Succession Type (SG Specialist)	-1.764	0.930	-1.896	0.0609
Mean body wight of seed disperser	-0.148	0.105	-1.406	0.1629
Leaf toughness	0.8380	0.564	1.486	0.1406
Wood density gravity	-1.530	0.823	-1.858	0.0663
Specific leaf area	**-0.940**	**0.358**	**-2.626**	**0.0101***
Leaf greenness	2.045	1.689	1.211	0.2290
Maximum plant height	**0.710**	**0.301**	**2.356**	**0.0205***
Leaf area	**0.732**	**0.226**	**3.236**	**0.0017** **

Abbreviations: SG, Second growth/Secondary forest; OG, Old growth

Supplementary Table 5. Results of GLS and PGLS models with the coefficient, standard error, t-value, and p-value for each trait. $p < 0.05$, * $p < 0.01$, ** $p < 0.001$, ***. Bold character indicates traits significantly related to seed volume of liana species.

**GLS models**				
	**Coefficient**	**Std.Error**	**t-value**	**p-value**
(Intercept)	**7.065**	**0.248**	**28.481**	**0.0000*****
Mean body wight of seed disperser	0.374	0.331	1.132	0.2671
Leaf toughness	0.558	0.995	0.561	0.5791
Specific leaf area	0.476	1.315	0.362	0.7198
Leaf greenness	1.884	2.439	0.773	0.4462
Leaf area	0.735	0.595	1.234	0.2273
**PGLS models**				
(Intercept)	**6.812**	**1.662**	**4.097**	**0.0003*****
Mean body wight of seed disperser	0.346	0.178	1.939	0.0626
Leaf toughness	1.169	0.810	1.442	0.1600
Specific leaf area	0.230	0.868	0.265	0.7928
Leaf greenness	1.457	1.782	0.818	0.4204
Leaf area	0.603	0.362	1.666	0.1069

Supplementary Figures

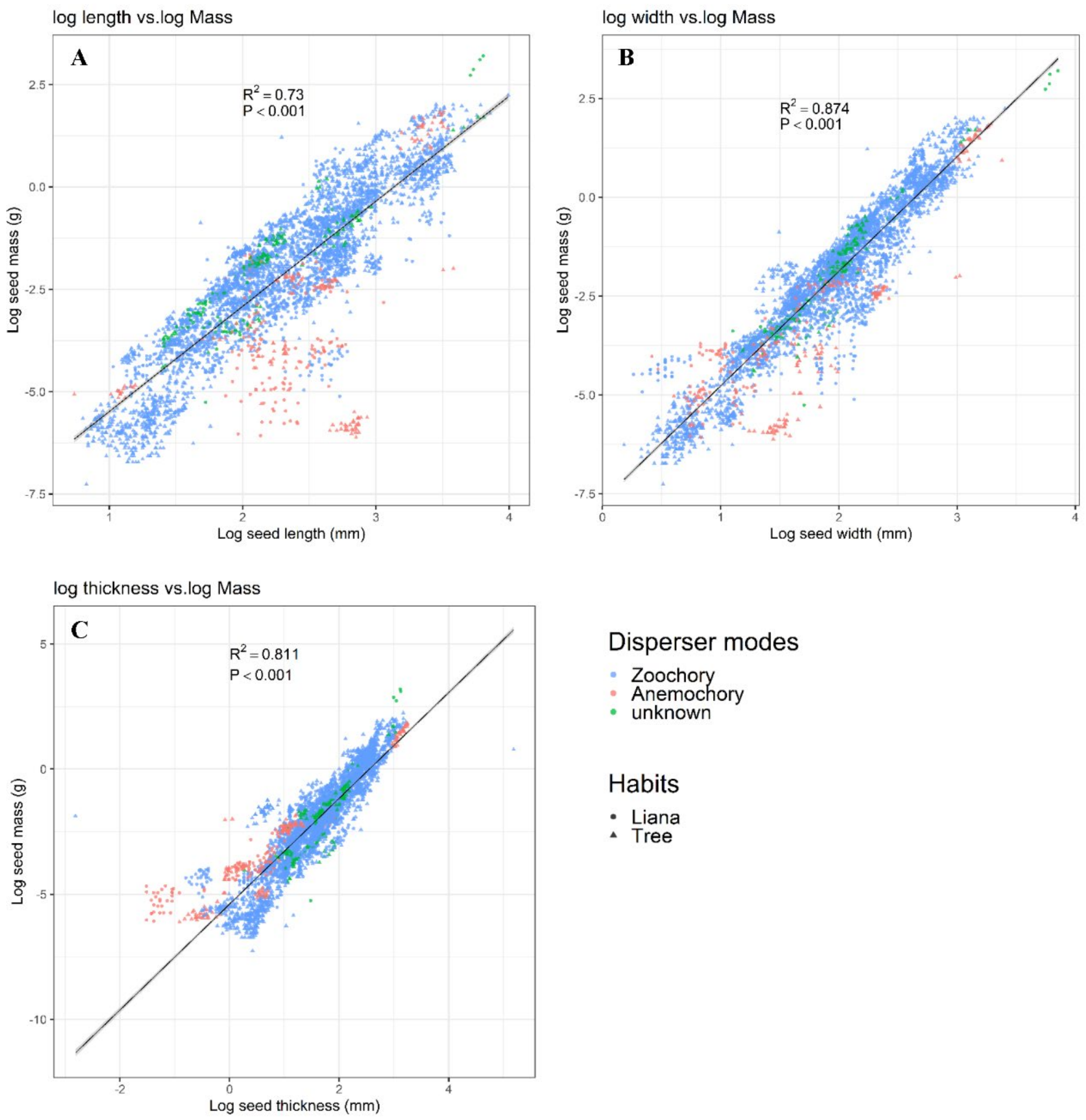

Supplementary Figure 1. Relationships between seed mass and volume–related traits in wind and animal dispersed tree and liana species. The black solid line shows the linear regression fit for the trend and the confidence interval (grey envelopes) (n = 5,219).

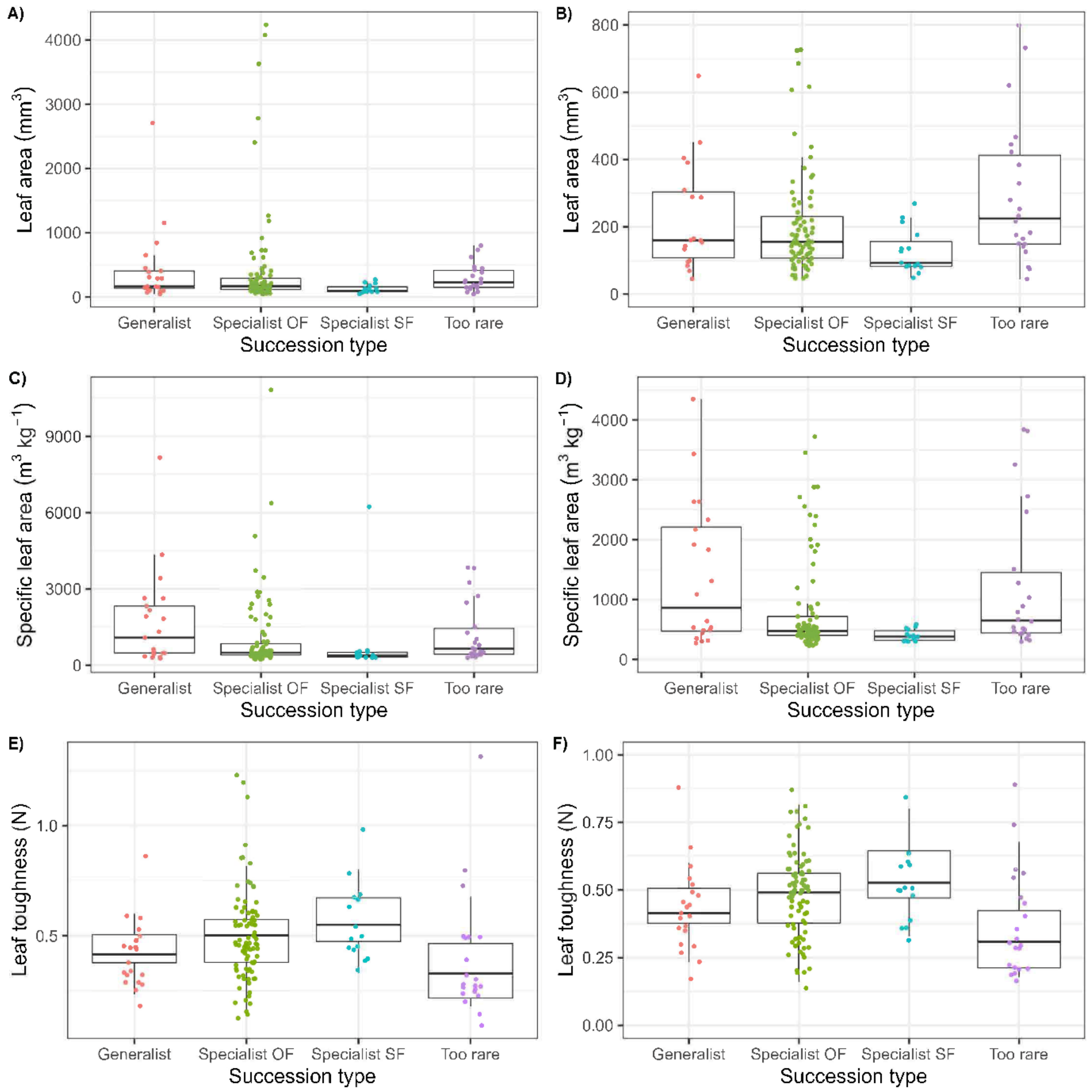

Supplementary Figure 2. Boxplots showing the leaf trait distributions in each succession niche. Boxplots were paired by the ones with the range of all values (left panel) and the ones limiting the maximum values for better visualization on the y-axis (right). Leaf traits are leaf area (A, B), specific leaf area (C, D) and leaf toughness (E, F). Note that the box shows a median as a thick horizontal band, both ends of the box denoted an interquartile range and any points outside the lines are shown as outliers.

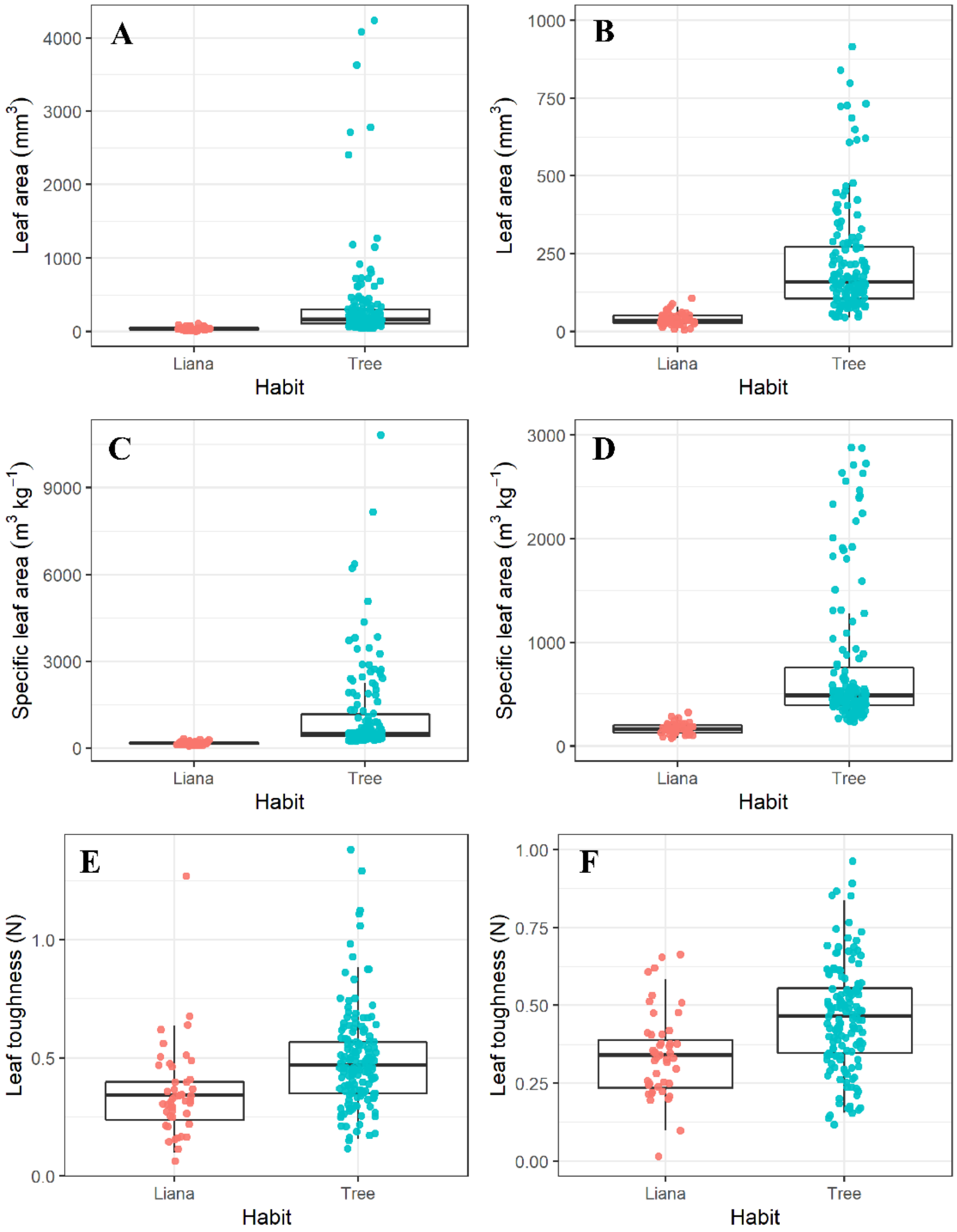

Supplementary Figure 3. Boxplots of the leaf trait distributions comparing between tree and liana species. Boxplots were paired by the ones with the range of all values (left panel) and the ones limiting the maximum values for better visualization on the y-axis (right). Leaf traits are leaf area (A, B), specific leaf area (C, D) and leaf toughness (E, F). Note that the box shows the median result as a thick horizontal band, the ends of the box denote the interquartile range and any points outside these values are shown as outliers (136 tree and 30 liana species).
