# Supplementary figures and images for: Seed Size Variation of Trees and Lianas in a Tropical Forest of Southeast Asia: Allometry, Phylogeny, and Seed Trait - Plant Functional Trait Relationships
Source: Front Plant Sci. 2022 May 17;13:852167. doi: 10.3389/fpls.2022.852167 (PMC9165448; doi:10.3389/fpls.2022.852167)

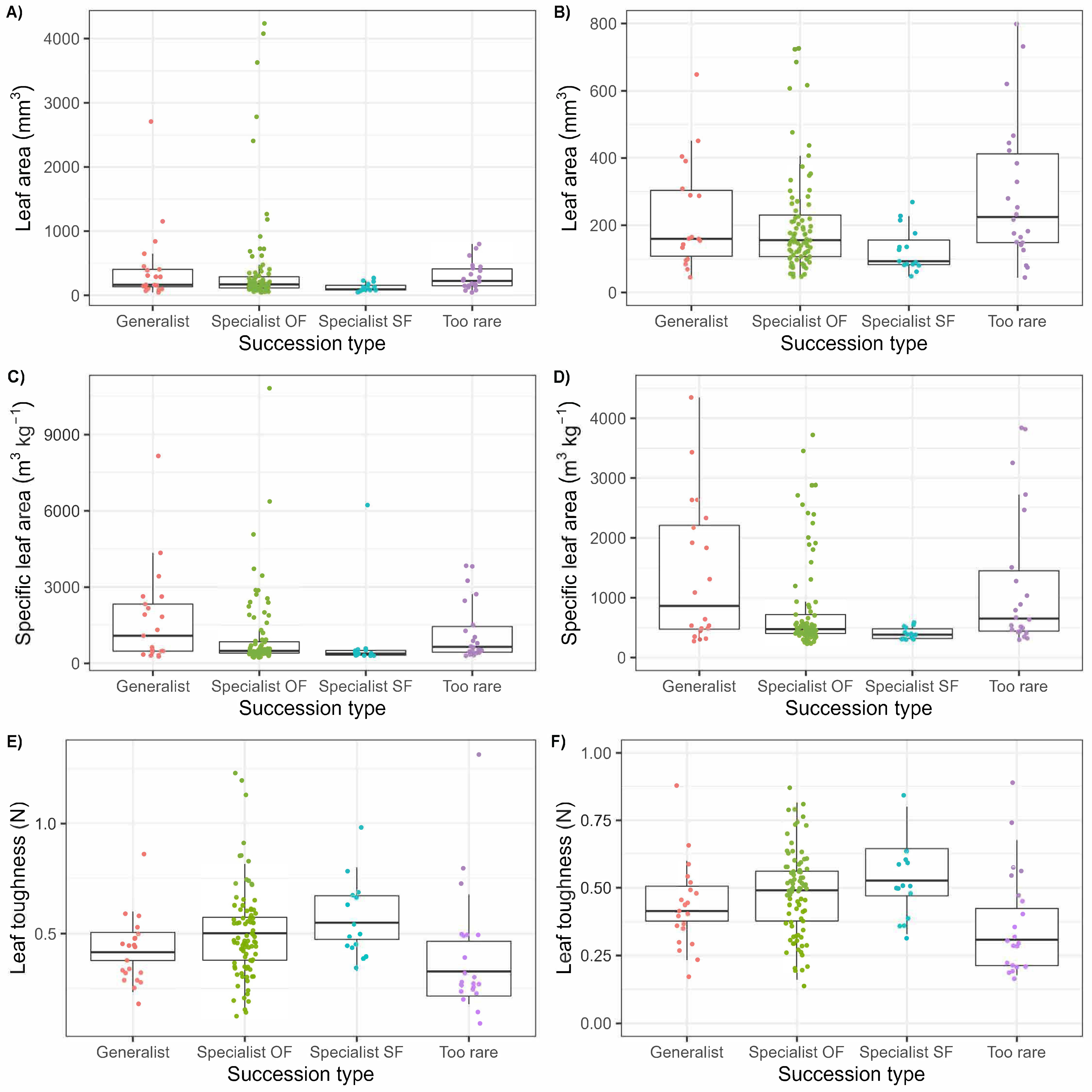

Supplement: Supplementary file 2 [file Image_1.tif]
